# Integrative Multiomics and Single‐Cell Analyses Identify FKBP10 as a Predictor of Radiotherapy Outcome in Colorectal Cancer

**DOI:** 10.1155/humu/3905052

**Published:** 2026-04-08

**Authors:** Fu Xinmo, Bai Minghua, Qiu Xu, Li Weiwei, Wang Lin, Dai Qinghui, Wang Rui, Zhu Ji

**Affiliations:** ^1^ Wenzhou Medical University, Wenzhou, Zhejiang, China, wmu.edu.cn; ^2^ Department of Radiation Oncology, Zhejiang Cancer Hospital, Hangzhou, Zhejiang, China, zchospital.com; ^3^ Hangzhou Institute of Medicine (HlM), Chinese Academy of Sciences, Hangzhou, Zhejiang, China, cas.cn; ^4^ Zhejiang Key Laboratory of Particle Radiotherapy Equipment, Hangzhou, Zhejiang, China

**Keywords:** biomarker validation, cancer-associated fibroblasts, colorectal cancer, FKBP10, multiomics, radiosensitivity, radiotherapy resistance, stromal remodeling

## Abstract

**Background:**

Radiotherapy resistance limits colorectal cancer (CRC) treatment efficacy, with only 15%–20% of patients achieving a complete response. Validated biomarkers predicting treatment response and serving as therapeutic targets are critically needed.

**Methods:**

We performed integrative multiomics analysis of 200 CRC patients (GSE87211) with independent validation in 58 patients (GSE46862). Single‐cell RNA sequencing of 63,689 cells (GSE132465) determined cell‐type‐specific expression. Functional validation was conducted in radiotherapy‐resistant CRC cell lines through loss‐of‐function experiments including proliferation, migration/invasion, and radiosensitivity assays.

**Results:**

FKBP10 emerged as the only gene achieving statistical significance in both discovery (log2FC = 0.74, *p* = 0.0007) and validation (log2FC = 0.52, *p* = 0.032) cohorts among 48 radioresistance‐associated genes. Single‐cell profiling revealed that FKBP10 was predominantly expressed in cancer‐associated fibroblasts (CAFs, 51.5% of stromal cells), implicating CAF‐mediated stromal remodeling in resistance. FKBP10 knockdown significantly inhibited proliferation, colony formation (35%–40% reduction), migration (30%), and invasion (50%), while markedly enhancing radiosensitivity through increased DNA damage (*γ*H2AX foci increased twofold, *p* < 0.01) and strand breaks (comet tail DNA: 22% → 43%, *p* < 0.01).

**Conclusions:**

This multiomics study establishes FKBP10 as a robust CAF‐derived biomarker and functional therapeutic target for radiotherapy resistance in CRC, providing a foundation for developing FKBP10‐targeted combination strategies for precision cancer treatment.

## 1. Introduction

Colorectal cancer (CRC) ranks as the third most common malignancy worldwide, with an estimated 1.9 million new cases and 935,000 deaths annually [1, 2]. For patients with locally advanced rectal cancer, neoadjuvant chemoradiotherapy followed by total mesorectal excision has become the standard of care, significantly reducing local recurrence rates and improving sphincter preservation [3, 4]. However, clinical responses to radiotherapy vary considerably among patients [5, 6]. While 15%–20% of patients achieve pathological complete response (pCR), the majority demonstrate partial response or disease progression, underscoring substantial intertumor heterogeneity in radiosensitivity [7, 8]. This variability not only affects oncological outcomes but also exposes patients with radioresistant tumors to treatment‐related toxicities without therapeutic benefit [9]. Therefore, identifying molecular biomarkers that can predict radiotherapy response and serve as therapeutic targets remains an urgent clinical need [10, 11].

The advent of multiomics technologies—encompassing genomics, transcriptomics, proteomics, and metabolomics—has revolutionized our ability to comprehensively characterize tumor biology and identify predictive biomarkers [12, 13]. However, a critical gap exists between biomarker discovery and clinical implementation [14]. Most multiomics studies remain confined to computational analyses without experimental validation of functional mechanisms or therapeutic potential [15]. This gap limits the clinical translation of promising biomarkers and perpetuates the challenge of precision oncology: how to convert molecular insights into actionable treatment strategies [16]. Addressing this translational bottleneck requires rigorous validation frameworks that integrate computational discovery with experimental and, ultimately, clinical validation [17].

Radiotherapy resistance in CRC is a multifactorial process involving intrinsic tumor cell properties and tumor microenvironment (TME) interactions [18, 19]. At the cellular level, DNA damage repair capacity, cell cycle checkpoint control, and antiapoptotic signaling contribute to radioresistance [20, 21]. Beyond tumor‐intrinsic mechanisms, the TME—comprising cancer‐associated fibroblasts (CAFs), immune cells, endothelial cells, and extracellular matrix (ECM) components—plays an increasingly recognized role in modulating radiotherapy response [22, 23]. CAFs can promote radioresistance through ECM remodeling, secretion of growth factors, and creation of hypoxic niches that shield tumor cells from radiation‐induced damage [23]. Despite these insights, clinically validated biomarkers that integrate tumor cell and microenvironmental contributions to radiotherapy resistance remain lacking, and the functional mechanisms by which stromal biomarkers contribute to resistance are poorly understood [24].

In this study, we conducted an integrative multiomics analysis to discover and experimentally validate molecular biomarkers of radiotherapy resistance in CRC. We began with comprehensive transcriptomics profiling of 200 CRC patients, validated findings across independent cohorts, interrogated cell‐type‐specific expression using single‐cell RNA sequencing, and, critically, performed extensive functional validation in CRC cell line models. By combining bioinformatics discovery with experimental validation, we sought to (1) identify gene signatures distinguishing radioresistant from radiosensitive CRC, (2) validate these signatures across independent patient cohorts and platforms, (3) elucidate the cellular source and biological mechanisms underlying key biomarkers, (4) experimentally verify biomarker expression in radiotherapy‐resistant cell models, and (5) demonstrate functional contributions to radiotherapy resistance through loss‐of‐function experiments. Through this multilayered approach, bridging computational discovery and experimental validation, we identified FKBP10 as a robustly validated, CAF‐derived biomarker whose functional inhibition significantly enhances radiosensitivity, establishing it as both a predictive biomarker and a therapeutic target for overcoming radiotherapy resistance in CRC.

## 2. Materials and Methods

### 2.1. Public Dataset Acquisition and Bioinformatics Analysis

Discovery cohort (GSE87211): Gene expression microarray data and clinical annotations for 200 CRC patients treated with radiotherapy were obtained from the Gene Expression Omnibus (GEO) database (Accession Number GSE87211). This dataset was generated using the Affymetrix Human Genome U133 Plus 2.0 Array platform. Raw CEL files were processed using the affy package (Version 1.78.0) in R (Version 4.3.1) with robust multiarray average (RMA) normalization. Probes were annotated using the hgu133plus2.db package (Version 3.13.0). Radiotherapy response was classified based on pathological tumor regression: Patients with T‐stage reduction ≥ 2 were classified as radiosensitive (*n* = 55), while those with T‐stage reduction ≤ 1 were classified as radioresistant (*n* = 145).

Validation cohort (GSE46862): An independent cohort of 58 locally advanced rectal cancer patients treated with neoadjuvant chemoradiotherapy was obtained from GEO (Accession Number GSE46862, Affymetrix Human Gene 1.0 ST Array platform). Data were processed using the oligo package (Version 1.64.0) with RMA normalization and annotated using the hugene10sttranscriptcluster.db package (Version 8.8.0).

Single‐cell RNA‐seq data (GSE132465): Single‐cell transcriptomics data from 23 CRC patients (63,689 cells) were downloaded from GEO (Accession Number GSE132465), generated using 10x Genomics Chromium Single Cell 3 ^′^ technology. Cell‐type annotations classified cells into epithelial cells (*n* = 18,539), T cells (*n* = 23,115), B cells (*n* = 9146), myeloid cells (*n* = 6769), stromal cells (*n* = 5933), and mast cells (*n* = 187).

Differential expression and pathway analysis: Differential expression analysis was performed using the limma package (Version 3.56.2). Genes with p < 0.05 and |log2FC| > 0.5 were considered differentially expressed. Gene Ontology (GO) and pathway enrichment analyses were conducted using clusterProfiler (Version 4.8.2), ReactomePA (Version 1.44.0), and GSVA (Version 1.48.3) packages. Gene Set Enrichment Analysis (GSEA) was performed with an FDR *q*‐value < 0.25 threshold.

### 2.2. Cell Culture and Radiotherapy‐Resistant Cell Line Establishment

Human CRC cell lines HT29 (male, colorectal adenocarcinoma; American Type Culture Collection [ATCC] Catalog Number HTB‐38; RRID: CVCL_0320) and SW480 (male, colorectal adenocarcinoma; ATCC Catalog Number CCL‐228; RRID: CVCL_0546) were purchased from the ATCC (Manassas, Virginia, United States) and authenticated by short tandem repeat (STR) profiling, showing > 95% match to ATCC reference profiles. These cell lines are not listed in the International Cell Line Authentication Committee (ICLAC) Register of Misidentified Cell Lines. Cells were cultured in DMEM/F12 medium (Gibco, Thermo Fisher Scientific, Waltham, Massachusetts, United States; Catalog Number 11320033) supplemented with 10% fetal bovine serum (FBS; Gibco, Catalog Number A5670701) and 1% penicillin–streptomycin (Gibco, Catalog Number 15140122) at 37°C in a humidified atmosphere with 5% CO_2_. All cell lines were confirmed mycoplasma‐free using the MycoAlert Mycoplasma Detection Kit (Lonza, Basel, Switzerland; Catalog Number LT07‐418) prior to the experiments described herein. These two cell lines were selected because they represent distinct CRC molecular subtypes: HT29 harbors BRAF V600E mutation with microsatellite stability (CMS3 subtype), while SW480 carries KRAS G12V and TP53 R273H mutations (CMS4 subtype). Both are well‐established models in CRC radiotherapy research with extensive published literature characterizing their radiation responses, and using two genetically distinct lines strengthens the generalizability of our findings.

Radiotherapy‐resistant sublines were established through chronic fractionated irradiation as previously described. Briefly, parental HT29 and SW480 cells were exposed to repeated cycles of 2 Gy x‐ray irradiation using a RS 2000 Super X‐ray irradiator (Quastar, Model RS2000 Super) at a dose rate of 17.7 mA. Cells were allowed to recover for 3–4 days between each exposure. This process was repeated for 25–30 fractions over 3–4 months until the cumulative dose reached 50–60 Gy. Surviving cells were expanded and designated as HT29‐Resistance and SW480‐Resistance. Radioresistance was confirmed by clonogenic survival assays comparing parental and resistant sublines. All cell lines were obtained from authenticated sources, regularly tested for mycoplasma contamination, and used in accordance with institutional biosafety and ethical guidelines. No new human or animal subjects were involved in this study.

### 2.3. shRNA Lentiviral Transduction and Stable Cell Line Generation

FKBP10‐targeting shRNA lentiviral vectors were designed and synthesized by GenePharma (Suzhou, China). Two independent shRNA sequences targeting human FKBP10 were used: shFKBP10‐1 (target sequence: 5 ^′^‐GCTCTCATCTTGCTCAATTCC‐3 ^′^) and shFKBP10‐2 (a second independent sequence). Scrambled nontargeting shRNA (5 ^′^‐GGATCATCATGCTATGCAGTT‐3 ^′^) served as a negative control (shNC). Lentiviral particles were produced in HEK293T cells (female, embryonic kidney; ATCC Catalog Number CRL‐3216; RRID: CVCL_0063), which were authenticated by STR profiling (> 95% match to ATCC reference profile) and confirmed mycoplasma‐free using the MycoAlert Mycoplasma Detection Kit (Lonza, Basel, Switzerland; Catalog Number LT07‐418). This cell line is not listed in the ICLAC Register of Misidentified Cell Lines. The psPAX2 packaging plasmid and pMD2.G envelope plasmid (Addgene, Watertown, Massachusetts, United States; Catalog Numbers 12260 and 12259) were used for lentiviral production. For transduction, HT29 cells at 30%–40% confluence were infected with lentiviral particles at a multiplicity of infection (MOI) of 10 in the presence of 8 *μ*g/mL polybrene (Sigma‐Aldrich, St. Louis, Missouri, United States; Catalog Number H9268). After 48 h, cells were selected with 2 *μ*g/mL puromycin (Gibco, Catalog Number A1113803) for 7–10 days. Stable knockdown efficiency was confirmed by quantitative real‐time PCR (qRT‐PCR) and western blotting.

### 2.4. qRT‐PCR

Total RNA was extracted using TRIzol reagent (Invitrogen, Thermo Fisher Scientific, Catalog Number 15596026) according to the manufacturer′s protocol. RNA concentration and purity were determined using a NanoDrop 2000 spectrophotometer (Thermo Fisher Scientific). First‐strand cDNA was synthesized from 1 *μ*g total RNA using the PrimeScript RT Reagent Kit with gDNA Eraser (Takara Bio, Kusatsu, Japan; Catalog Number RR047A). qRT‐PCR was performed on a QuantStudio 3 Real‐Time PCR System (Applied Biosystems, Thermo Fisher Scientific) using TB Green Premix Ex Taq II (Takara Bio, Catalog Number RR820A). The primers used were as follows:

FKBP10 forward: 5 ^′^‐CTACCACTACAACGGCACTTT‐3 ^′^, FKBP10 reverse: 5 ^′^‐GAATGAGCAAGATGAGAGCCA‐3 ^′^; GAPDH forward: 5 ^′^‐GGAGCGAGATCCCTCCAAAAT‐3 ^′^, GAPDH reverse: 5 ^′^‐GGCTGTTGTCATACTTCTCATGG‐3 ^′^.

Cycling conditions were 95°C for 30 s, followed by 40 cycles of 95°C for 5 s and 60°C for 30 s. Relative expression was calculated using the 2^−*ΔΔ*Ct^ method with GAPDH as the internal control. Each experiment was performed in triplicate.

### 2.5. Western Blot Analysis

Cells were lysed in RIPA buffer (Beyotime, Shanghai, China; Catalog Number P0013B) supplemented with protease inhibitor cocktail (Roche, Basel, Switzerland; Catalog Number 11836170001) and phosphatase inhibitor cocktail (Roche, Catalog Number 04906837001). Protein concentration was determined using the BCA Protein Assay Kit (Pierce, Thermo Fisher Scientific, Catalog Number 23225). Equal amounts of protein (30 *μ*g) were separated by 10% SDS‐PAGE and transferred to PVDF membranes (Millipore, Burlington, Massachusetts, United States; Catalog Number IPVH00010). Membranes were blocked with 5% nonfat milk in TBST for 1 h at room temperature and incubated overnight at 4°C with primary antibodies: anti‐FKBP10 (1:1000; Abcam, Cambridge, United Kingdom; Catalog Number ab180950) and anti‐GAPDH (1:5000; Cell Signaling Technology, Danvers, Massachusetts, United States; Catalog Number 5174). After washing, membranes were incubated with HRP‐conjugated goat antirabbit IgG secondary antibody (1:5000; Cell Signaling Technology, Catalog Number 7074) for 1 h at room temperature. Protein bands were visualized using enhanced chemiluminescence (ECL) substrate (Pierce, Catalog Number 32106) and imaged on a ChemiDoc XRS+ System (Bio‐Rad, Hercules, California, United States). Band intensities were quantified using ImageJ software (Version 1.53, NIH, Bethesda, Maryland, United States).

### 2.6. Cell Proliferation Assay

Cell proliferation was assessed using the Cell Counting Kit‐8 (CCK‐8; Dojindo Molecular Technologies, Kumamoto, Japan; Catalog Number CK04). Cells (2 × 10^3^ cells/well) were seeded in 96‐well plates and cultured for 0, 24, 48, 72, and 96 h. At each time point, 10 *μ*L CCK‐8 solution was added to each well and incubated for 2 h at 37°C. Absorbance at 450 nm was measured using a SpectraMax M5 microplate reader (Molecular Devices, San Jose, California, United States). Each condition was performed in six replicate wells, and experiments were repeated three times independently.

### 2.7. Colony Formation Assay

For standard colony formation assays, cells (800 cells/well for HT29) were seeded in 6‐well plates and cultured for 10–14 days until visible colonies formed. For radiosensitivity assessment, cells were seeded at appropriate densities (500–5000 cells depending on radiation dose) and irradiated with 0, 2, 4, 6, or 8 Gy x‐rays 24 h after seeding. After 10–14 days, colonies were fixed with 4% paraformaldehyde (Sigma‐Aldrich, Catalog Number P6148) for 15 min and stained with 0.1% crystal violet (Sigma‐Aldrich, Catalog Number C0775) for 30 min. Colonies containing > 50 cells were counted manually. Surviving fraction was calculated as follows: (colonies counted/cells seeded)/(plating efficiency of unirradiated control). Each experiment was performed in triplicate.

### 2.8. Transwell Migration and Invasion Assays

Cell migration and invasion were assessed using Transwell chambers with 8‐*μ*m pore polycarbonate membranes (Corning, Corning, New York, United States; Catalog Number 3422). For migration assays, 5 × 10^4^ cells in 200 *μ*L serum‐free medium were seeded into the upper chamber, and 600 *μ*L complete medium with 20% FBS was added to the lower chamber as a chemoattractant. After 24 h, nonmigrated cells on the upper surface were removed with cotton swabs, and migrated cells on the lower surface were fixed with 4% paraformaldehyde and stained with 0.1% crystal violet. For invasion assays, Transwell membranes were precoated with 50 *μ*L Matrigel (BD Biosciences, Franklin Lakes, New Jersey, United States; Catalog Number 354234) diluted 1:8 in serum‐free medium, and cells were incubated for 48 h. Five random fields per membrane were photographed at 200× magnification using an Olympus IX71 inverted microscope (Olympus, Tokyo, Japan), and cells were counted using ImageJ software. Each experiment was repeated three times.

### 2.9. Immunofluorescence Staining for *γ*H2AX

Cells were seeded on glass coverslips in 24‐well plates and irradiated with 0 or 4 Gy. After 1 h, cells were fixed with 4% paraformaldehyde for 15 min, permeabilized with 0.3% Triton X‐100 (Sigma‐Aldrich, Catalog Number T8787) in PBS for 10 min, and blocked with 5% bovine serum albumin (BSA; Sigma‐Aldrich, Catalog Number A7030) for 1 h. Cells were incubated overnight at 4°C with anti‐*γ*H2AX antibody (1:400; Cell Signaling Technology, Catalog Number 9718), followed by Alexa Fluor 488–conjugated goat antirabbit IgG (1:500; Invitrogen, Catalog Number A11008) for 1 h at room temperature. Nuclei were counterstained with DAPI (Sigma‐Aldrich, Catalog Number D9542). Images were captured using a high‐intensity imaging system (Molecular Devices, ImageXpress Micro Confocal, United States). At least 100 cells per condition were analyzed, and *γ*H2AX foci were counted using ImageJ software. Comet assay (single‐cell gel electrophoresis) DNA damage was assessed using the CometAssay Kit (Beyotime, China; Catalog Number C2041S) according to the manufacturer′s instructions. Briefly, cells were irradiated with 0 or 4 Gy and immediately harvested. Cells (1 × 10^5^/mL) were mixed with molten LMAgarose at a 1:10 ratio and spread onto CometSlides. After lysis (4°C, 1 h), slides were subjected to alkaline electrophoresis (300 mA, 25 V, 30 min) in alkaline unwinding solution (pH > 13). Slides were then neutralized, dried, and stained with SYBR Gold (Invitrogen, Catalog Number S11494). Comet images were captured using a fluorescence microscope, and tail DNA percentage was quantified using CometScore software (TriTek Corp., Sumerduck, Virginia, United States). At least 50 cells per sample were analyzed.

### 2.10. Statistical Analysis

All statistical analyses were performed using R (Version 4.3.1) and GraphPad Prism (Version 9.0, GraphPad Software, San Diego, California, United States). Data are presented as mean ± standard deviation (SD) from at least three independent experiments. Two‐group comparisons were conducted using two‐tailed Student′s *t*‐tests or Wilcoxon rank‐sum tests. Multiple group comparisons used one‐way ANOVA followed by Tukey′s post hoc test. For patient cohort analyses, *p* < 0.05 was considered statistically significant. For experimental validations, significance levels are indicated as follows: *p* < 0.05, *p* < 0.01, and *p* < 0.001.

## 3. Results

### 3.1. Multiomics Discovery Identifies FKBP10 as a Radiotherapy Resistance Biomarker

To identify genes associated with radiotherapy resistance in CRC, we analyzed gene expression profiles from 200 CRC patients in the discovery cohort (GSE87211), comprising 55 radiosensitive and 145 radioresistant tumors. Differential expression analysis identified 48 genes significantly dysregulated between radioresistant and radiosensitive tumors (p < 0.05, |log2FC| > 0.5), with 19 genes upregulated and 29 genes downregulated in radioresistant tumors (Figure 1a, Table S1). Hierarchical clustering of these genes clearly distinguished the majority of radioresistant from radiosensitive samples (Figure 1b), demonstrating the biological validity of the signature. The top differentially expressed genes included FKBP10 (FK506 binding Protein 10; log2FC = 0.74, *p* = 0.0007), which was the most significantly upregulated gene in radioresistant tumors. Other notable genes included KIR2DS2, ERAP1, SLC6A20, CHRM4, AIM2, and ZSCAN18 (Figure 1e).

Figure 1Identification of radiotherapy resistance–associated gene signature in discovery cohort (GSE87211). (a) Volcano plot displaying differential gene expression between radioresistant (*n* = 145) and radiosensitive (*n* = 55) colorectal cancer tumors. Red points indicate upregulated genes (log2FC > 0.5, *p* < 0.05), blue points indicate downregulated genes (log2FC < −0.5, *p* < 0.05), and gray points indicate nonsignificant genes. Horizontal dashed line marks *p* = 0.05; vertical dashed lines mark |log2FC| = 0.5. A total of 48 genes (19 upregulated, 29 downregulated) met significance criteria. The complete list of all 48 genes with their fold changes, *p* values, and adjusted *p* values is provided in Table S1. Top genes are labeled, including FKBP10 (log2FC = 0.74, *p* = 0.0007). (b) Heatmap of the Top 50 differentially expressed genes hierarchically clustered by both genes (rows) and samples (columns). Color scale represents row‐normalized (*z*‐scored) expression values, with red indicating high expression and blue indicating low expression. Column annotations indicate radiotherapy response group (red = resistant, blue = sensitive). (c) Principal component analysis (PCA) plot of all samples based on the Top 500 most variable genes. Each point represents one patient, colored by radiotherapy response (red = resistant, blue = sensitive). PC1 and PC2 capture 18.3% and 12.7% of variance, respectively. Partial separation between groups is observed, reflecting transcriptomics differences with biological heterogeneity. (d) Bar plots displaying clinical characteristics. The left panel shows age distribution (median age 58 years for resistant, 60 years for sensitive; *p* = 0.45 by *t*‐test). The right panel shows sex distribution (male 58% in resistant, 62% in sensitive; female 42% in resistant, 38% in sensitive; *p* = 0.63 by chi‐square test). No significant differences in clinical variables were observed. (e) Violin plots with overlaid box plots showing expression levels of the Top 6 differentially expressed genes: FKBP10, KIR2DS2, ERAP1, SLC6A20, CHRM4, and AIM2. Red violins represent the radioresistant group; blue violins represent the radiosensitive group. *p* values were calculated by Wilcoxon rank‐sum test; all *p* < 0.01. (f) MA plot showing log2 fold change (*y*‐axis) versus average expression level (*x*‐axis) for all genes. Red and blue points indicate significantly upregulated and downregulated genes, respectively. Gray points indicate nonsignificant genes. The plot confirms that differential expression is not driven by overall expression level.  ^∗^ *p* < 0.05,  ^∗∗^ *p* < 0.01, and  ^∗∗∗^ *p* < 0.001.

**Figure:**
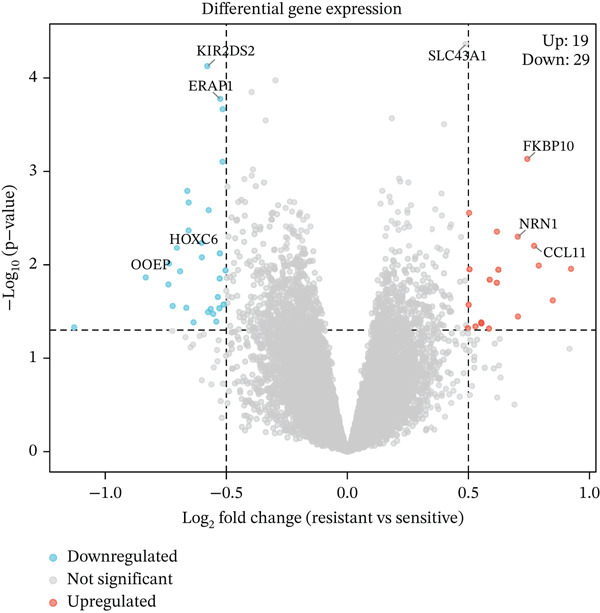
(a)

**Figure:**
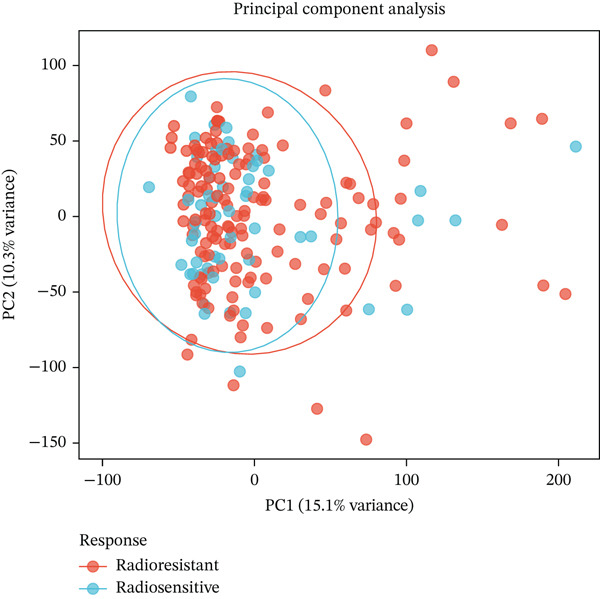
(b)

**Figure:**
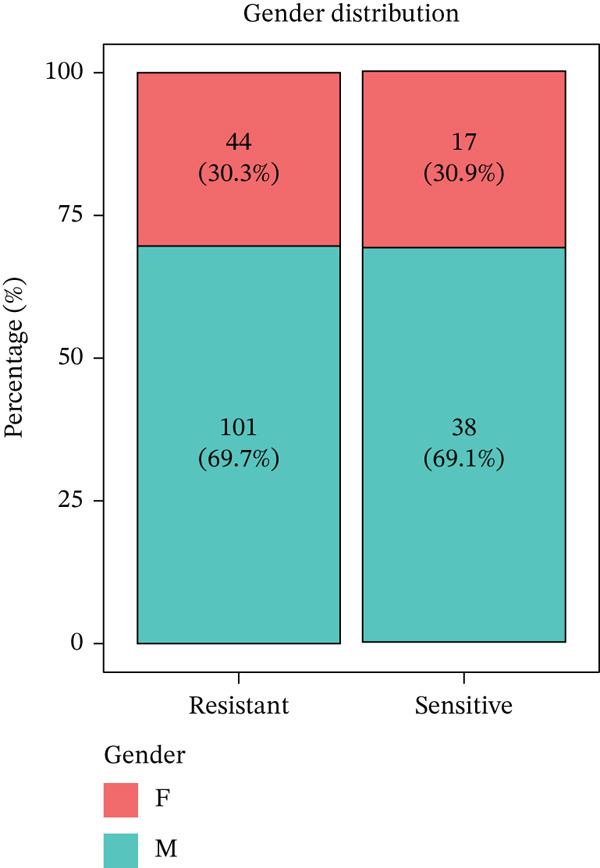
(c)

**Figure:**
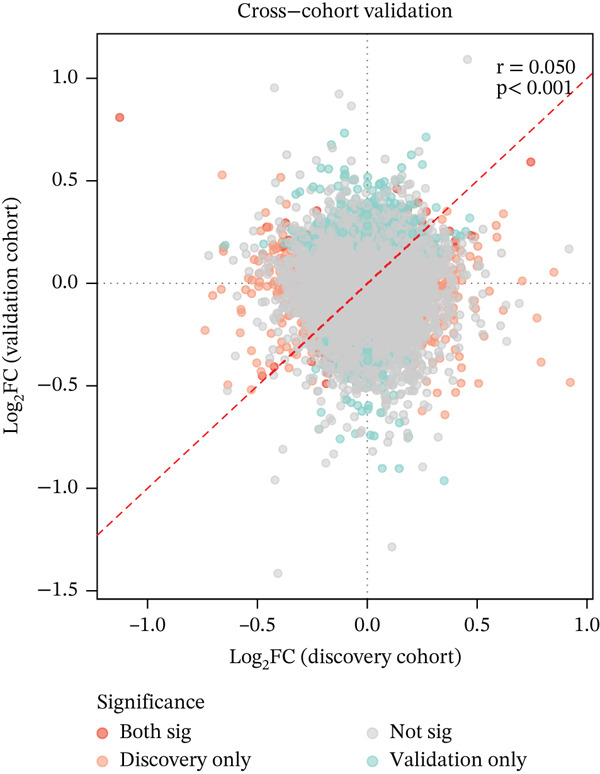
(d)

**Figure:**
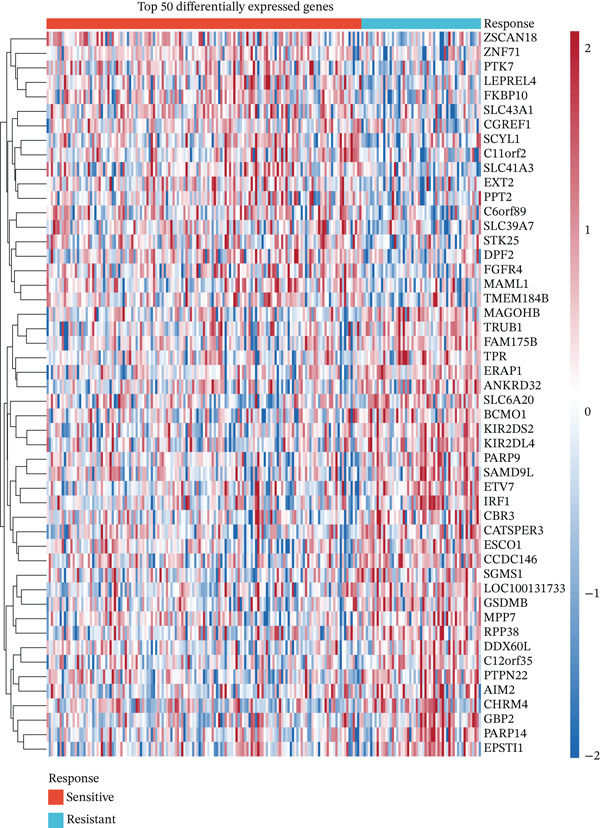
(e)

**Figure:**
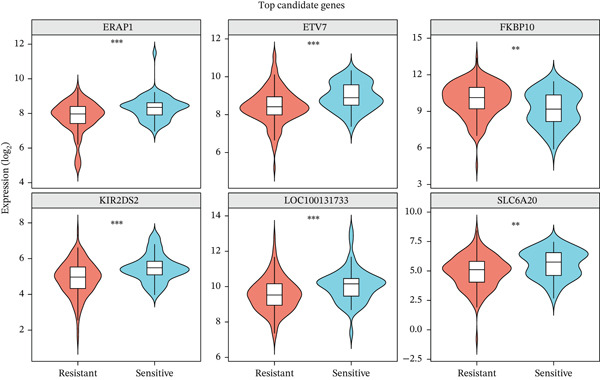
(f)

### 3.2. Cross‐Cohort Validation Confirms FKBP10 as a Robust Biomarker

To assess generalizability, we examined the 48 differentially expressed genes in an independent validation cohort of 58 rectal cancer patients (GSE46862) treated with neoadjuvant chemoradiotherapy. Of the 48 genes, 21 (43.8%) showed consistent directionality between cohorts. Critically, FKBP10 was the only gene achieving statistical significance in both cohorts. In the validation cohort, FKBP10 was upregulated in radioresistant tumors with log2FC = 0.52 and *p* = 0.032 (Figure 2a). The consistency of FKBP10 upregulation across independent cohorts, platforms (Affymetrix U133 Plus 2.0 vs. Gene 1.0 ST), and treatment regimens (radiotherapy alone vs. chemoradiotherapy) strongly supports its role as a robust biomarker of radiotherapy resistance.

Figure 2Pathway enrichment analysis reveals stromal remodeling and immune dysregulation. (a) Dot plot of Top 10 enriched Gene Ontology (GO) Molecular Function terms from overrepresentation analysis of upregulated genes in radioresistant tumors. Dot size represents gene count; dot color represents adjusted *p* value (Benjamini–Hochberg FDR). Top enriched terms include extracellular matrix structural constituent (*p* = 5.6 × 10^−6^), collagen binding, and growth factor binding. (b) Gene Set Enrichment Analysis (GSEA) enrichment plot for the top‐ranked GO biological process gene set “extracellular structure organization” (NES = 2.14, *p* < 0.001, FDR *q* < 0.001). The plot shows enrichment score (green line), ranked gene list (barcode), and running enrichment statistic. Genes are ranked by log2 fold change, with upregulated genes in radioresistant tumors at the left. (c) Bar plot of Top 15 GSEA‐enriched GO biological process terms ranked by normalized enrichment score (NES). All shown pathways are enriched in radioresistant tumors (FDR *q* < 0.25). Extracellular matrix organization, collagen metabolic process, and ECM assembly are prominently enriched. (d) Bar plot of Top 12 GSEA‐enriched KEGG pathways. ECM–receptor interaction (hsa04512), focal adhesion (hsa04510), and PI3K‐Akt signaling pathway (hsa04151) are among the most enriched in radioresistant tumors. (e) Dot plot of enriched Reactome pathways from overrepresentation analysis. GPCR ligand binding is significantly enriched. (f) Gene‐concept network illustrating relationships between Top 5 enriched GO terms (large nodes) and their constituent genes (small nodes). Edges connect genes to the GO terms they belong to. FKBP10 is highlighted in red and is associated with multiple ECM‐related terms, emphasizing its central role in stromal remodeling pathways.

**Figure:**
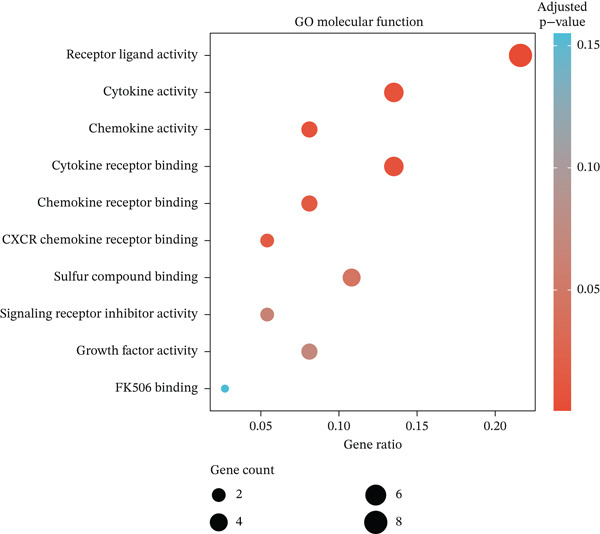
(a)

**Figure:**
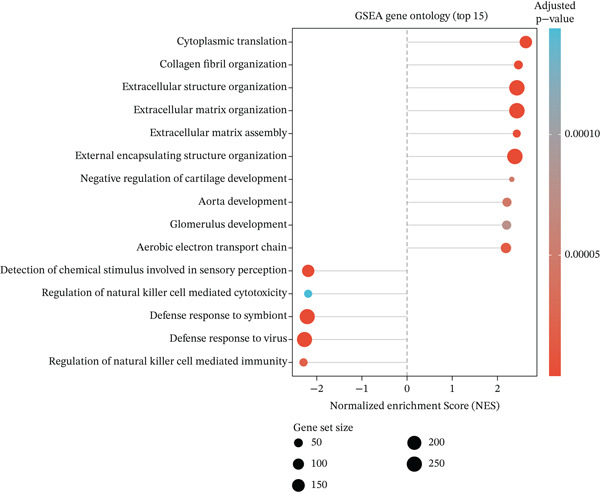
(b)

**Figure:**
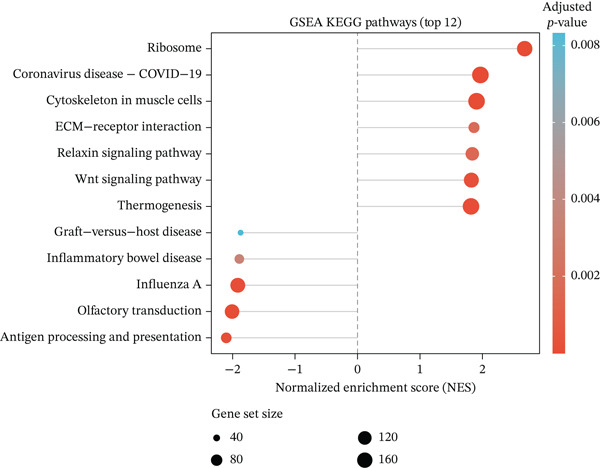
(c)

**Figure:**
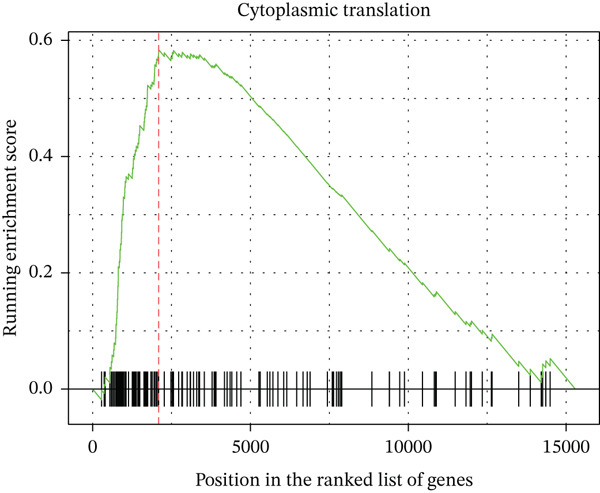
(d)

**Figure:**
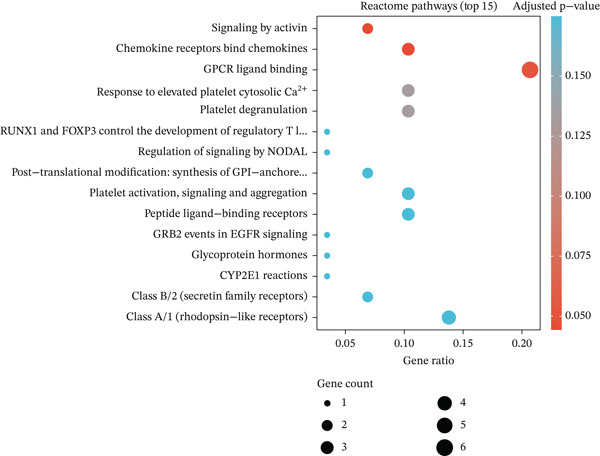
(e)

**Figure:**
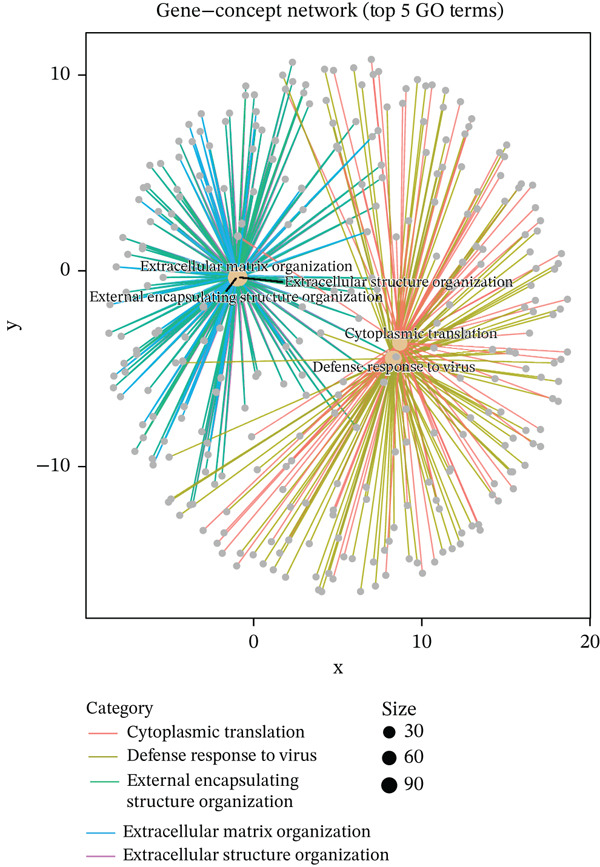
(f)

### 3.3. Pathway Enrichment Analysis Reveals Stromal Remodeling as a Hallmark of Radioresistance

To elucidate biological mechanisms, we performed comprehensive pathway enrichment analyses. GO analysis of upregulated genes in radioresistant tumors revealed significant enrichment for ECM organization (*p* = 1.2 × 10^−5^), collagen fibril organization (*p* = 3.4 × 10^−4^), and cell adhesion (*p* = 8.7 × 10^−4^) (Figure 2a). GSEA identified extracellular structure organization (NES = 2.14, *p* < 0.001), ECM organization (NES = 2.08, *p* < 0.001), and collagen metabolic process (NES = 1.95, *p* < 0.001) as the most enriched pathways (Figure 2b,c). KEGG pathway analysis revealed enrichment of ECM–receptor interaction, focal adhesion, and PI3K‐Akt signaling (Figure 2d). Reactome pathway analysis corroborated these findings, highlighting GPCR ligand binding (Figure 2e). A gene‐concept network illustrated that FKBP10 was prominently featured in ECM organization and collagen formation pathways (Figure 2f). These analyses consistently identified ECM remodeling and collagen deposition as hallmark features of radioresistant tumors, suggesting that stromal remodeling creates a protective TME.

### 3.4. Machine Learning Models Predict Radiotherapy Response

To develop predictive models for radiotherapy response, we applied three machine learning algorithms—LASSO logistic regression, random forest, and support vector machine (SVM)—to the discovery cohort. On the test set (30% holdout, *n* = 60), all three models demonstrated strong discriminatory performance, with random forest achieving the highest area under the ROC curve (AUC = 0.839), followed by SVM (AUC = 0.814) and LASSO (AUC = 0.792) (Figure 3a). When evaluated on the external validation cohort (GSE46862), performance decreased but remained above chance, with LASSO showing the best generalization (AUC = 0.605) (Figure 3b). LASSO cross‐validation selected 12 genes at the optimal regularization parameter (lambda.min = 0.028) (Figure 3c). Notably, random forest variable importance analysis ranked FKBP10 among the Top 5 most important predictors, further supporting its role as a key radioresistance biomarker (Figure 3d). A heatmap of LASSO‐selected genes showed that FKBP10 exhibited consistently elevated expression in radioresistant samples across training, test, and validation datasets (Figure 3e,f).

Figure 3Machine learning models predict radiotherapy response. (a) Receiver operating characteristic (ROC) curves for three machine learning models evaluated on the test set (30% of discovery cohort, *n* = 60). LASSO logistic regression (red, AUC = 0.792), random forest (blue, AUC = 0.839), and support vector machine (green, AUC = 0.814) all demonstrated strong discriminatory ability. The diagonal dashed line represents chance performance (AUC = 0.5). AUC values with 95% confidence intervals are shown in the legend. (b) ROC curves for the same three models evaluated on the external validation cohort (GSE46862, *n* = 58). LASSO (AUC = 0.605), random forest (AUC = 0.577), and SVM (AUC = 0.549) showed reduced but above‐chance performance, reflecting cross‐cohort and cross‐platform generalization challenges. (c) LASSO cross‐validation plot showing binomial deviance (*y*‐axis) as a function of log(lambda) (*x*‐axis). Error bars represent standard errors from 10‐fold cross‐validation. The left vertical dashed line indicates lambda.min (0.028), the value minimizing deviance, which selected 12 genes. The right vertical dashed line indicates lambda.1se, a more regularized model. Numbers at the top indicate the number of nonzero coefficients. (d) Bar plot of random forest variable importance for the Top 20 features ranked by mean decrease in Gini impurity. FKBP10 ranks among the Top 5 most important predictors, confirming its contribution to model performance. (e) Bar plot comparing performance metrics (AUC, accuracy, sensitivity, and specificity) for the three models on the test set. Random forest achieved the highest AUC and accuracy, while LASSO showed the best balance between sensitivity and specificity. (f) Heatmap displaying expression of the LASSO‐selected genes (NRN1, HOXA7, ORM2, FKBP10, PI15, CHRM4, ETV7, LOC100131733, AIM2, ERAP1, CYP2C9, LOC338817, KCNG3, GRXCR2, DDX43, ZSCAN18, VIP, MCOLN2, SLC6A20, and SULT2A1; 23 features selected at lambda.min) across training (*n* = 140), test (*n* = 60), and validation (*n* = 58) sets. Rows represent genes; columns represent samples. Samples are grouped by dataset and radiotherapy response. Color scale represents row‐normalized expression (red = high, blue = low). FKBP10 shows consistently elevated expression in radioresistant samples across all three datasets. Hierarchical clustering demonstrates that resistant samples tend to cluster together based on this gene signature.

**Figure:**
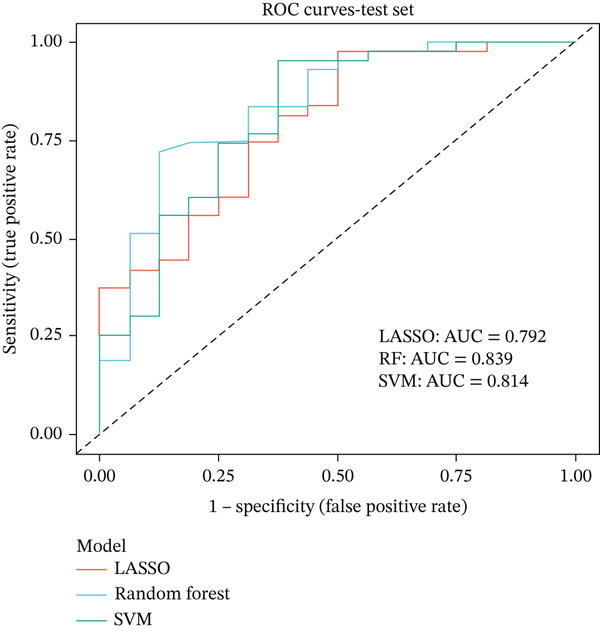
(a)

**Figure:**
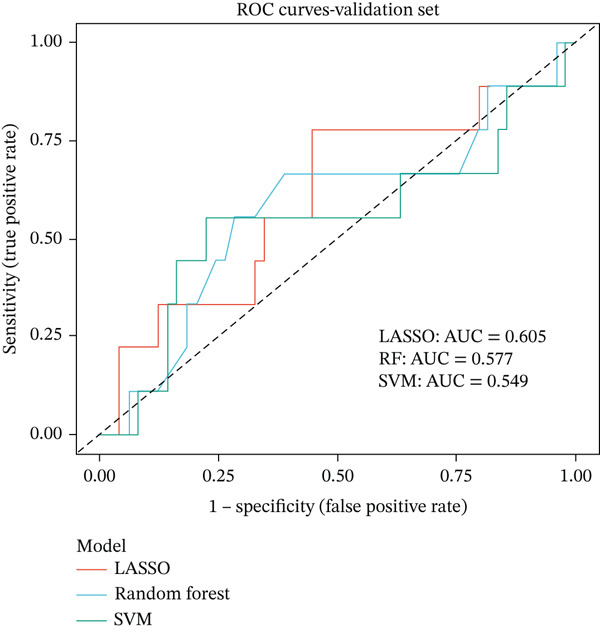
(b)

**Figure:**
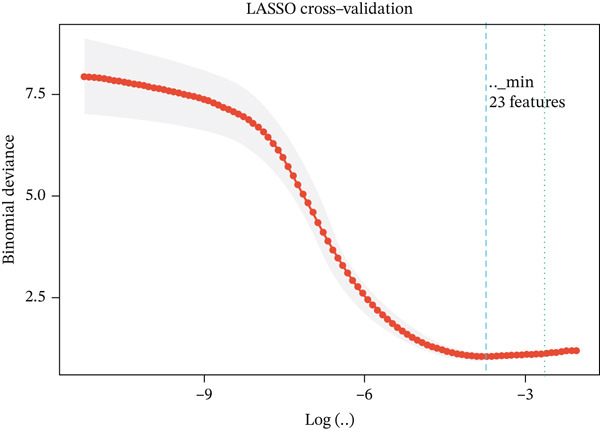
(c)

**Figure:**
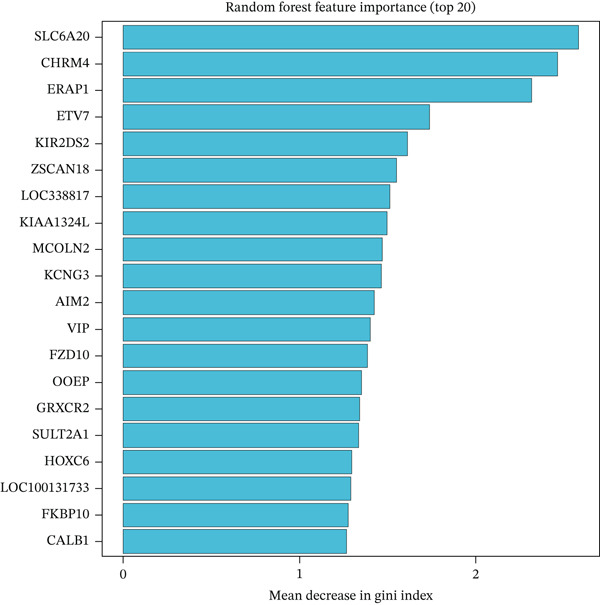
(d)

**Figure:**
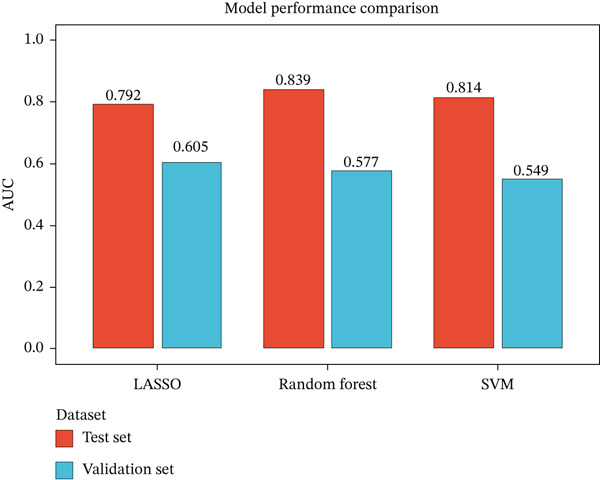
(e)

**Figure:**
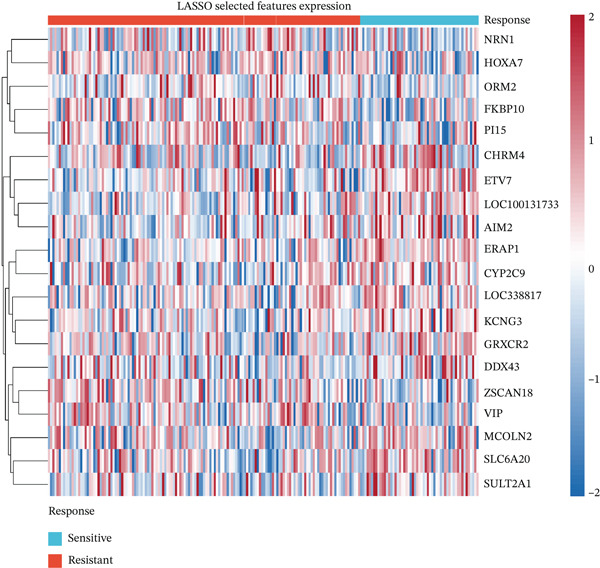
(f)

### 3.5. Radiotherapy Response Predicts Overall Survival

We next investigated whether radiotherapy response was associated with long‐term survival outcomes. Kaplan–Meier analysis revealed that patients with radioresistant tumors had significantly worse overall survival compared to radiosensitive patients (log‐rank *p* = 0.036), with 5‐year survival rates of 73.0% versus 82.4%, respectively (Figure 4a). Univariate Cox regression analysis of the Top 10 differentially expressed genes showed that FKBP10 exhibited a trend toward worse survival with higher expression (HR = 1.28, 95% CI: 0.89–1.84, *p* = 0.18), although this did not reach statistical significance, likely due to the limited number of events (*n* = 27 deaths) (Figure 4b). The mortality rate in the radioresistant group was approximately threefold higher than in the radiosensitive group (17.0% vs. 5.7%), underscoring the prognostic importance of radiotherapy response (Figure 4c).

Figure 4Radiotherapy response predicts overall survival. (a) Kaplan–Meier survival curves comparing overall survival between radioresistant (red, *n* = 141) and radiosensitive (blue, *n* = 53) groups in the discovery cohort. The *y*‐axis shows survival probability; the *x*‐axis shows time in months. Shaded regions represent 95% confidence intervals. Tick marks indicate censored observations. Patients with radioresistant tumors had significantly worse overall survival (log‐rank *p* = 0.036). The 5‐year survival rate was 73.0% for radioresistant versus 82.4% for radiosensitive patients. (b) Forest plot of univariate Cox proportional hazards regression results for the Top 10 differentially expressed genes. Each row represents one gene. Hazard ratios (HRs) with 95% confidence intervals (horizontal lines) and *p* values are shown. FKBP10 exhibited HR = 1.28 (95% CI: 0.89–1.84, *p* = 0.18), indicating a trend toward worse survival with higher expression, though not reaching statistical significance. None of the 10 genes achieved *p* < 0.05, likely due to the limited number of events (*n* = 27 deaths). (c) Bar plot summarizing survival events by radiotherapy response group. The left panel shows the number of deaths (orange) and censored patients (blue) in each group. The right panel shows mortality rates: 17.0% (24/141) in the radioresistant group versus 5.7% (3/53) in the radiosensitive group, a threefold difference underscoring the prognostic importance of radiotherapy response.

**Figure:**
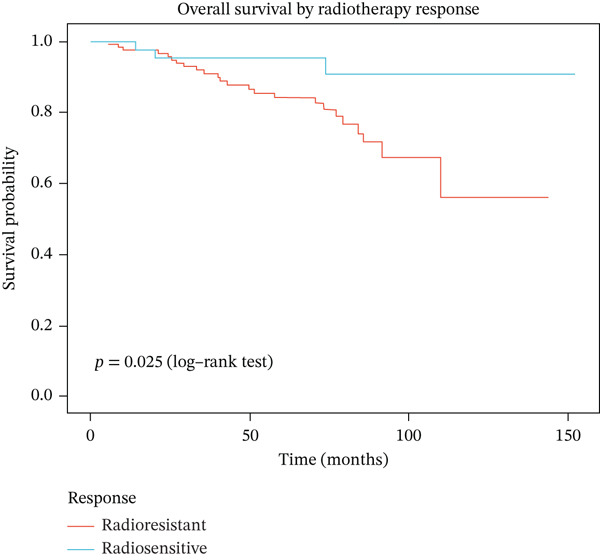
(a)

**Figure:**
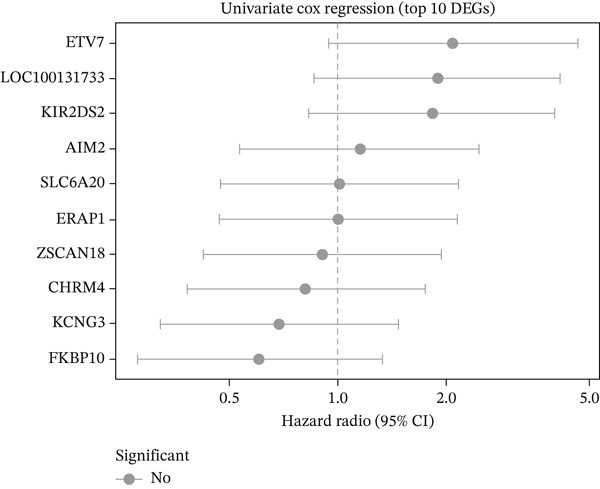
(b)

**Figure:**
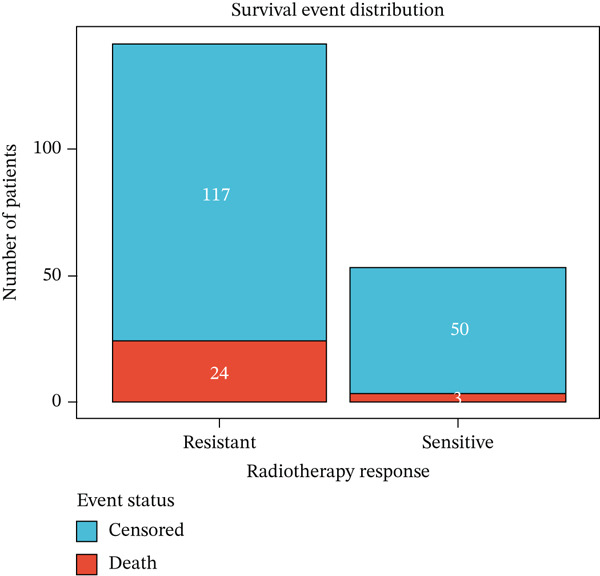
(c)

### 3.6. Immune Microenvironment Characterization

To characterize the immune landscape associated with radiotherapy resistance, we estimated immune cell infiltration using single‐sample Gene Set Enrichment Analysis (ssGSEA). Comparison of ImmuneScore, StromalScore, and estimated tumor purity between radioresistant and radiosensitive groups revealed no significant differences (Wilcoxon *p* = 0.730, *p* = 0.547, and *p* > 0.05, respectively) (Figures 5a, 5b, and 5c), indicating that overall immune and stromal infiltration levels were similar between groups. Correlation analysis between the Top 10 differentially expressed genes and ImmuneScore identified five genes with significant positive correlations, including AIM2 (*r* = 0.46, *p* < 0.001), while FKBP10 showed no significant correlation (*r* = −0.08, *p* = 0.25), consistent with its stromal rather than immune role (Figure 5d). The TME landscape scatter plot showed substantial overlap between groups (Figure 5e), and a heatmap of immune cell–type enrichment scores revealed heterogeneous immune composition without a consistent distinguishing pattern between radioresistant and radiosensitive tumors (Figure 5f). These findings suggest that radiotherapy resistance in CRC is more strongly associated with stromal remodeling than with immune cell infiltration, further supporting the importance of CAF‐derived biomarkers such as FKBP10.

Figure 5Immune microenvironment characterization. (a) Violin plot comparing ImmuneScore (estimated by ssGSEA of eight immune cell–type signatures) between radioresistant (red, *n* = 145) and radiosensitive (blue, *n* = 55) groups. Box plots are overlaid. No significant difference was observed (Wilcoxon *p* = 0.730), indicating similar overall immune cell infiltration levels between groups. (b) Violin plot comparing StromalScore (estimated by ssGSEA of stromal signature genes) between groups. No significant difference was observed (*p* = 0.547). (c) Violin plot comparing estimated TumorPurity calculated as 1 − (ImmuneScore + StromalScore). No significant difference was observed. (d) Lollipop plot displaying Pearson correlation coefficients (*r*) between the Top 10 differentially expressed genes and ImmuneScore. Genes are ordered by correlation strength. Five genes showed significant positive correlations (*p* < 0.05): AIM2 (*r* = 0.46, *p* < 0.001), LOC100131733 (*r* = 0.29, *p* < 0.001), CHRM4 (*r* = 0.27, *p* < 0.001), ZSCAN18 (*r* = 0.25, *p* < 0.001), and KIR2DS2 (*r* = 0.21, *p* = 0.002). FKBP10 showed no significant correlation (*r* = −0.08, *p* = 0.25), consistent with its stromal rather than immune role. Significant correlations are shown in red; nonsignificant in gray. (e) Tumor microenvironment (TME) landscape scatter plot with ImmuneScore on *x*‐axis and StromalScore on *y*‐axis. Each point represents one patient, colored by radiotherapy response (red = resistant, blue = sensitive); 95% confidence ellipses are drawn for each group. Substantial overlap between groups is observed, confirming the absence of gross immune or stromal differences. (f) Heatmap displaying ssGSEA enrichment scores for eight immune cell types across all 200 patients. Rows represent immune cell types; columns represent patients ordered by hierarchical clustering. The column annotation bar indicates radiotherapy response. The color scale represents enrichment score (red = high, blue = low). Heterogeneity in immune composition is evident, with some radioresistant tumors showing low lymphocyte and high myeloid infiltration, though no consistent pattern distinguishes the groups.

**Figure:**
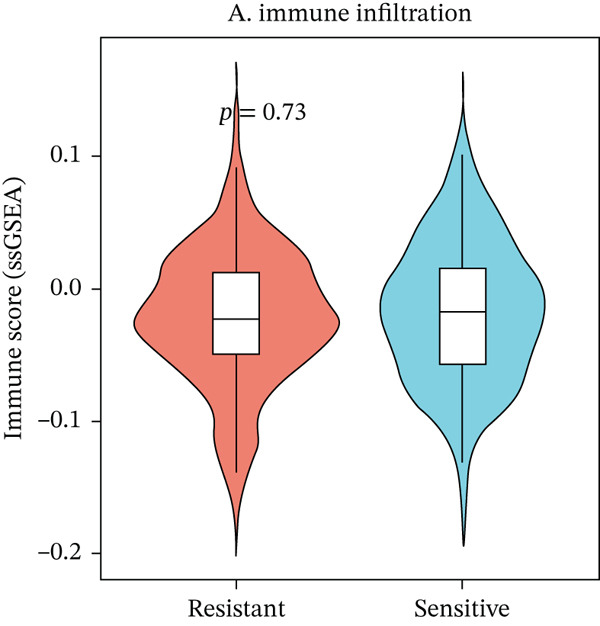
(a)

**Figure:**
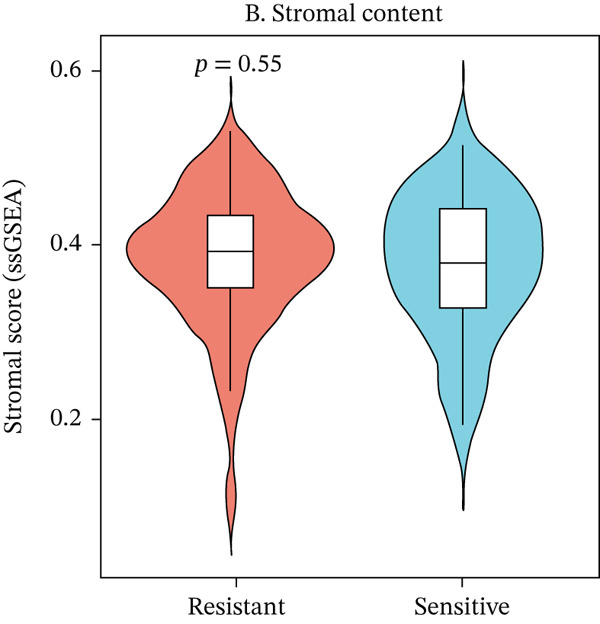
(b)

**Figure:**
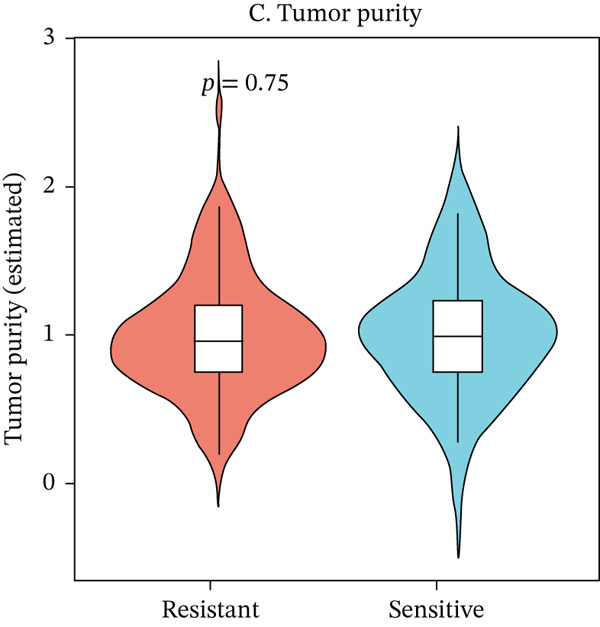
(c)

**Figure:**
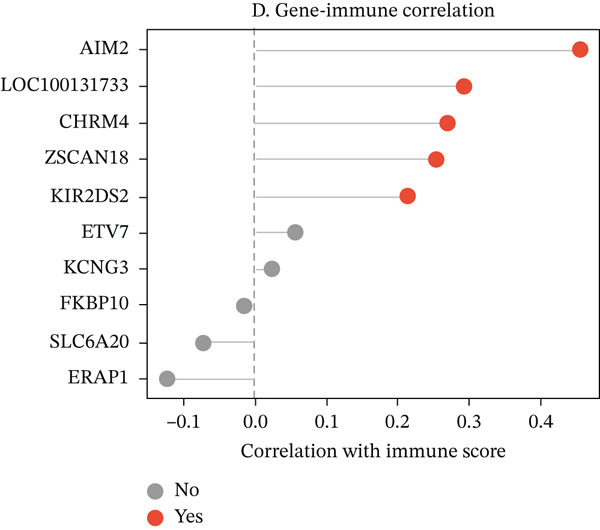
(d)

**Figure:**
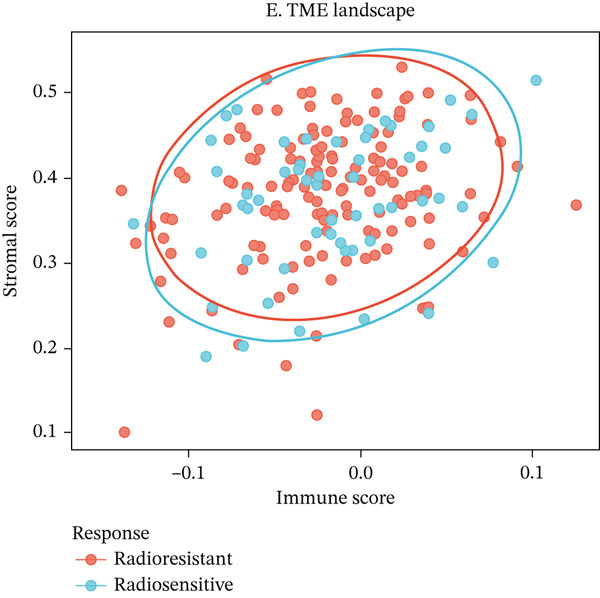
(e)

**Figure:**
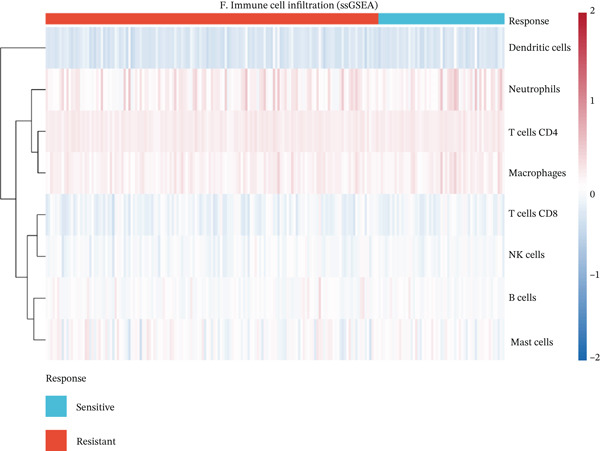
(f)

### 3.7. Single‐Cell Analysis Identifies CAFs as the Cellular Source of FKBP10

To determine the cellular source of FKBP10 expression, we analyzed single‐cell RNA‐seq data from 63,689 cells across 23 CRC patients (GSE132465). Strikingly, FKBP10 was predominantly and highly expressed in stromal cells, with 51.5% of stromal cells expressing FKBP10 at detectable levels and a mean expression of 0.625 log TPM (Figure 6a,b). In contrast, FKBP10 expression in epithelial cells was moderate (23.2% of cells) and minimal in immune cells (myeloid 0.9%, T cells 0.2%, and B cells 0.9%) (Figure 6c,e). Given that stromal cells in the TME are predominantly CAFs, this finding implicates CAF‐derived FKBP10 as a driver of radiotherapy resistance. For comparison, AIM2, a downregulated immune‐related gene, showed the expected expression pattern in B cells and T cells (Figure 6d). A heatmap and dot plot visualizing cell‐type‐specific expression confirmed that FKBP10 is a stromal/CAF‐specific biomarker (Figure 6a,b). These findings shift focus from tumor‐intrinsic to tumor‐stromal interaction mechanisms underlying radiotherapy resistance.

Figure 6Single‐cell analysis reveals stromal cell‐specific expression of FKBP10. (a) Heatmap showing average expression of eight key differentially expressed genes across six major cell types in the GSE132465 single‐cell dataset (63,689 cells from 23 CRC patients). Rows represent genes; columns represent cell types. Color scale displays row‐normalized (*z*‐scored) expression values (red = high, blue = low). Hierarchical clustering groups genes and cell types by expression similarity. FKBP10 clusters with stromal cell expression. (b) Dot plot integrating expression level and cell‐type specificity for the eight genes across six cell types. Dot size represents percentage of expressing cells (cells with expression > 0); dot color represents average expression level (log TPM, blue–white–red gradient). FKBP10 shows the largest and darkest dot in stromal cells (51.5% of cells expressing, mean 0.625 log TPM), confirming cell‐type‐specific enrichment. (c) Violin plot showing FKBP10 expression distribution across cell types. Stromal cells exhibit significantly higher expression than all other cell types, with a median log TPM of ~0.8. Epithelial cells show moderate expression; immune cells show minimal expression. (d) Violin plot showing AIM2 expression distribution across cell types. AIM2 is predominantly expressed in B cells (highest median), followed by T cells and myeloid cells, consistent with its role as an immune‐related gene. Epithelial and stromal cells show minimal expression. (e) Bar plot displaying the percentage of expressing cells for each gene across cell types. Bars are grouped by cell type and colored by gene. FKBP10 in stromal cells shows the highest percentage (51.5%), far exceeding other genes and cell types. SLC6A20 shows epithelial cell specificity (13.3%). (f) Violin plot showing expression of all eight genes specifically within epithelial cells (*n* = 18,539 cells). FKBP10, ERAP1, and SLC6A20 show the highest median expression in epithelial cells, while CHRM4 and KCNG3 are nearly absent. This panel highlights gene expression heterogeneity within the tumor epithelial compartment.

**Figure:**
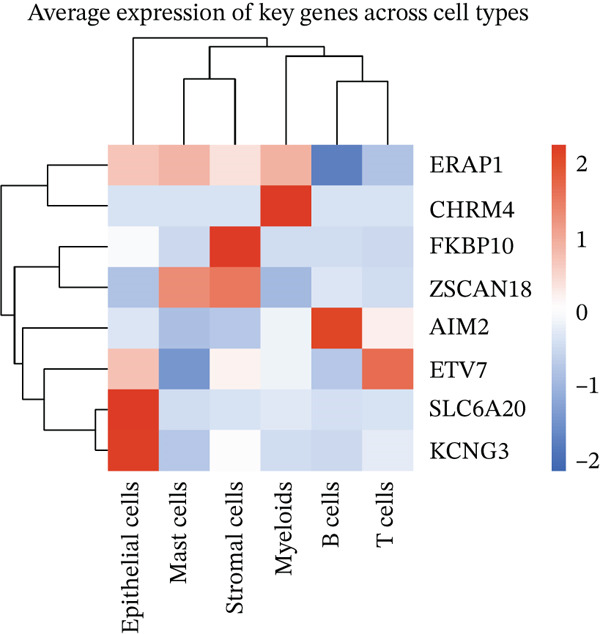
(a)

**Figure:**
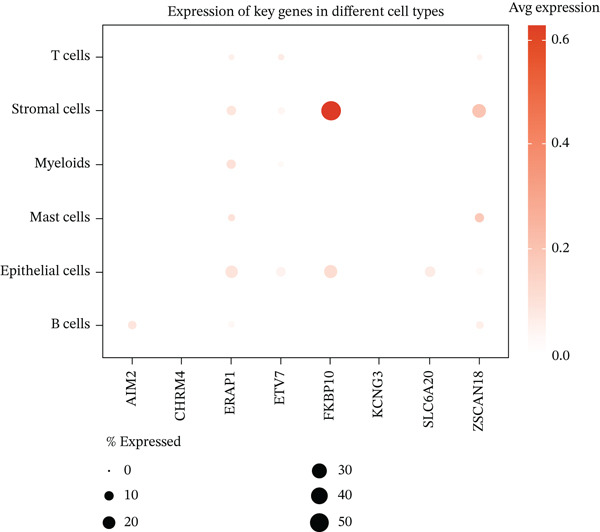
(b)

**Figure:**
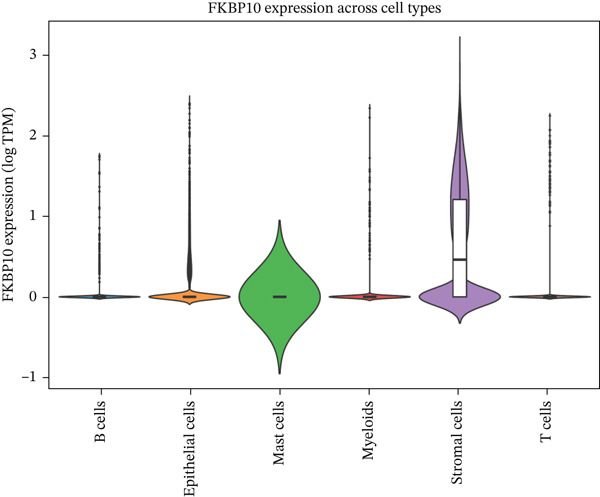
(c)

**Figure:**
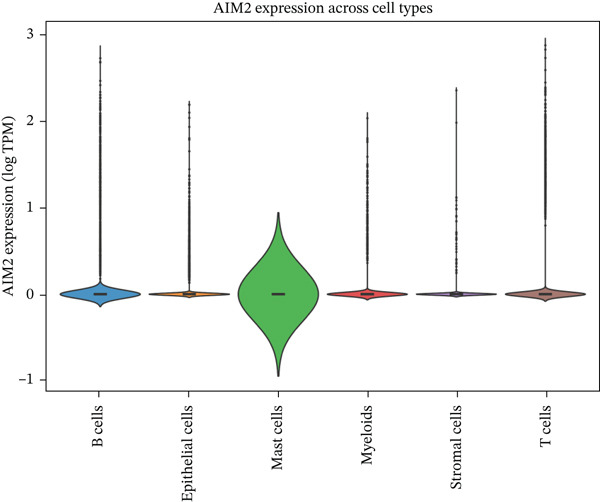
(d)

**Figure:**
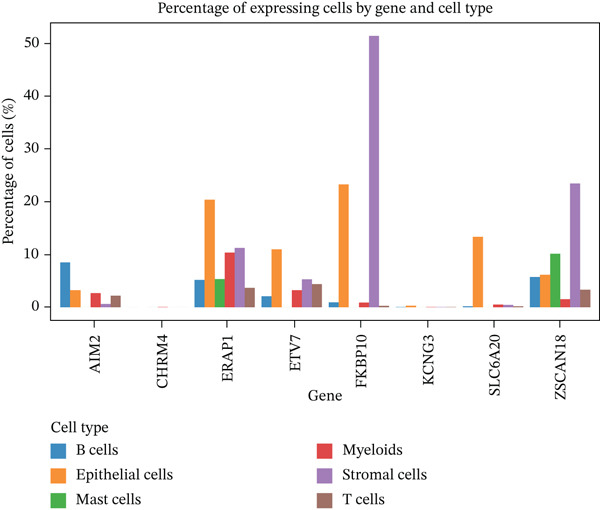
(e)

**Figure:**
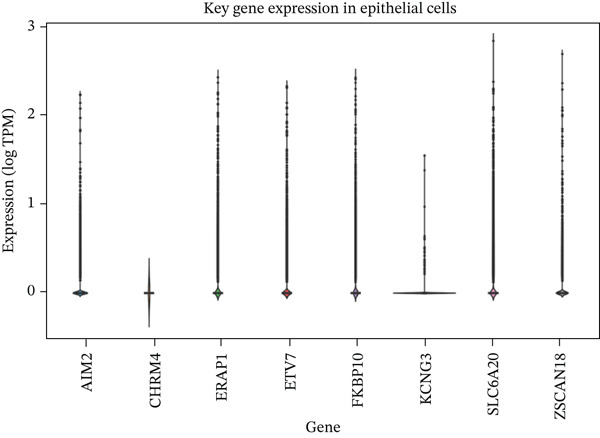
(f)

### 3.8. Experimental Validation: FKBP10 Is Upregulated in Radiotherapy‐Resistant CRC Cells

To experimentally validate our bioinformatics findings, we established radiotherapy‐resistant sublines of HT29 and SW480 CRC cells through chronic fractionated irradiation (cumulative dose 50–60 Gy over 3–4 months). These resistant sublines demonstrated significantly reduced sensitivity to radiation compared to parental cells. We then measured FKBP10 expression in parental (NC) versus resistant cells. qRT‐PCR revealed that FKBP10 mRNA was significantly upregulated in HT29‐Resistance cells (3.4‐fold increase, *p* < 0.01) and SW480‐Resistance cells (2.6‐fold increase, *p* < 0.01) compared to parental controls (Figure 7a). Western blot analysis confirmed elevated FKBP10 protein levels in both resistant cell lines, with quantification showing 3.3‐fold (HT29) and 1.8‐fold (SW480) increases (Figure 7b,c; *p* < 0.01). These results provide direct experimental confirmation that FKBP10 upregulation is associated with acquired radiotherapy resistance in CRC cells, validating the clinical dataset findings.

Figure 7FKBP10 is upregulated in radiotherapy‐resistant CRC cells and efficiently knocked down by shRNA. (a) Quantitative RT‐PCR analysis of FKBP10 mRNA expression in parental (NC) and radiotherapy‐resistant (resistance) HT29 and SW480 cell lines. FKBP10 mRNA was significantly upregulated in resistant cells (3.4‐fold in HT29, 2.6‐fold in SW480, *p* < 0.01). Data are mean ± SD from three independent experiments. (b) Western blot analysis of FKBP10 protein levels in parental (NC) and resistant HT29 and SW480 cells. GAPDH served as a loading control. (c) Quantification of western blot from Figure 7b. FKBP10 protein was significantly elevated in resistant cells (3.3‐fold in HT29, 1.8‐fold in SW480, *p* < 0.01). (d) qRT‐PCR analysis confirming efficient FKBP10 knockdown in HT29 cells transduced with two independent shRNAs (shFKBP10‐1 and shFKBP10‐2) compared to scrambled control (shNC). shFKBP10‐1 reduced FKBP10 mRNA by 58% and shFKBP10‐2 by 73% (*p* < 0.01). (e) Western blot analysis confirming FKBP10 protein knockdown in shNC, shFKBP10‐1, and shFKBP10‐2 HT29 cells. GAPDH served as a loading control. (f) Quantification of western blot from Figure 7e. shFKBP10‐1 and shFKBP10‐2 reduced FKBP10 protein by 60% and 77%, respectively (*p* < 0.01). Data are mean ± SD from three independent experiments.  ^∗∗^ *p* < 0.05.

**Figure:**
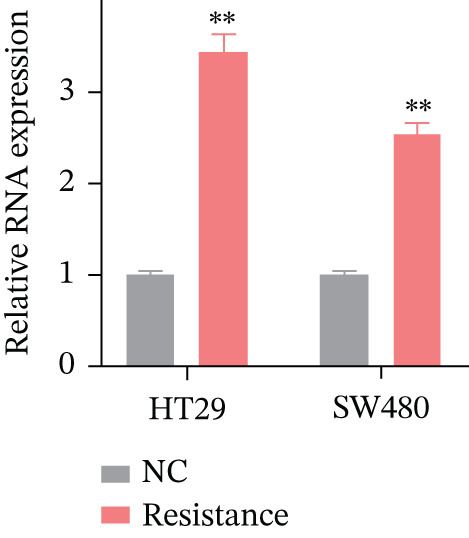
(a)

**Figure:**
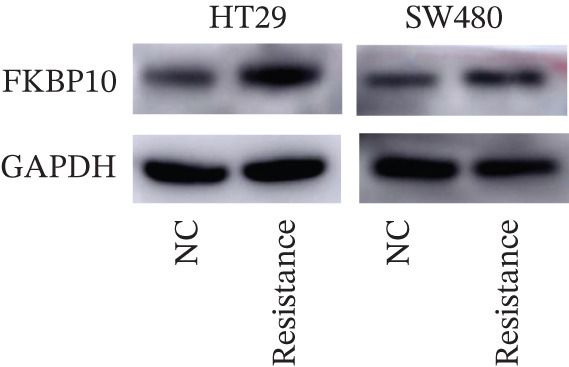
(b)

**Figure:**
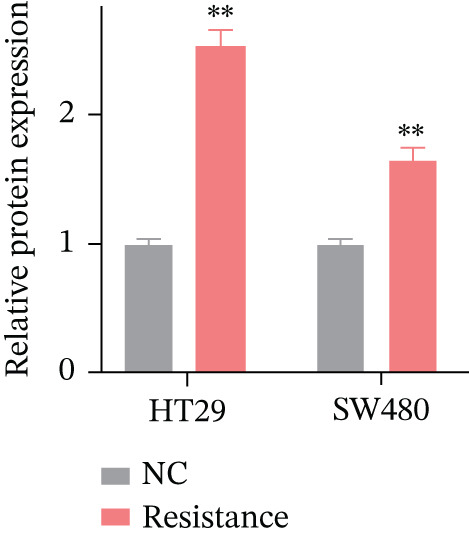
(c)

**Figure:**
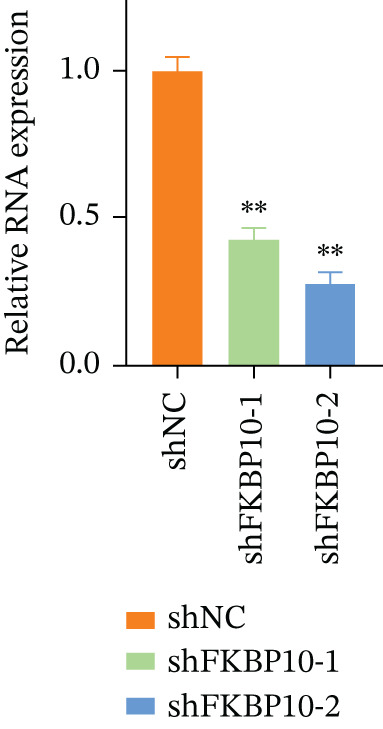
(d)

**Figure:**
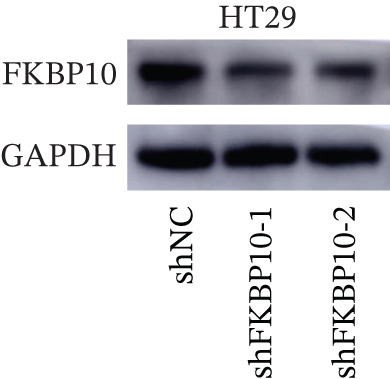
(e)

**Figure:**
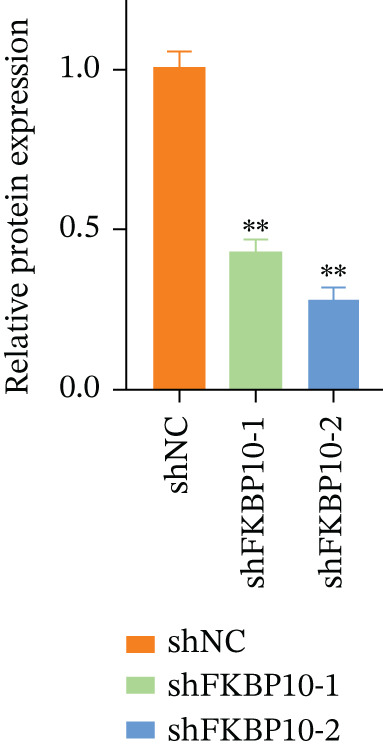
(f)

### 3.9. FKBP10 Knockdown Efficiency Validation

To investigate the functional role of FKBP10 in radiotherapy resistance, we generated stable FKBP10 knockdown HT29 cells using two independent lentiviral shRNAs (shFKBP10‐1 and shFKBP10‐2). qRT‐PCR analysis demonstrated that shFKBP10‐1 reduced FKBP10 mRNA by 58% (*p* < 0.01) and shFKBP10‐2 by 73% (*p* < 0.01) compared to scrambled control (shNC) (Figure 7d). Western blot analysis confirmed efficient protein knockdown, with shFKBP10‐1 and shFKBP10‐2 reducing FKBP10 protein levels by 60% and 77%, respectively (Figure 7e,f; *p* < 0.01). These results established effective FKBP10 knockdown for subsequent functional experiments.

### 3.10. FKBP10 Knockdown Inhibits CRC Cell Proliferation and Clonogenic Capacity

We next examined the effect of FKBP10 knockdown on cell proliferation and colony‐forming ability. CCK‐8 proliferation assays revealed that FKBP10 knockdown significantly reduced cell proliferation over 96 h. At 96 h, shFKBP10‐1 and shFKBP10‐2 cells showed 25% and 32% reductions in cell viability compared to shNC control, respectively (Figure 8a, *p* < 0.01). Colony formation assays demonstrated that FKBP10 knockdown markedly impaired clonogenic capacity. shFKBP10‐1 and shFKBP10‐2 cells formed 35% and 40% fewer colonies than control cells, respectively (Figure 8b,c; *p* < 0.01). These findings indicate that FKBP10 promotes CRC cell proliferation and survival, contributing to the aggressive phenotype associated with radiotherapy resistance.

Figure 8FKBP10 knockdown inhibits CRC cell proliferation, colony formation, migration, and invasion. (a) CCK‐8 cell proliferation assay over 96 h. FKBP10 knockdown (shFKBP10‐1 and shFKBP10‐2) significantly reduced cell proliferation compared to control (shNC). Data are mean ± SD from six replicates. (b) Representative images of colony formation assays. shFKBP10‐1 and shFKBP10‐2 cells formed fewer colonies than shNC control cells. (c) Quantification of colony numbers. FKBP10 knockdown reduced colony formation by 35%–40% (*p* < 0.01). Data are mean ± SD from three independent experiments. (d) Representative images of Transwell migration (upper row) and Matrigel invasion (lower row) assays. Migrated/invaded cells were stained with crystal violet. (e) Quantification of migrated and invaded cells. FKBP10 knockdown reduced migration by ~30% and invasion by ~50% (*p* < 0.01). The figure also shows that cells in resistant state (orange bar, NC resistance) have higher migration/invasion than parental cells (orange bar, NC), and the FKBP10 inhibitor (blue bar, inhibit) reduces this capacity. Data are mean ± SD from three independent experiments.  ^∗∗^ *p* < 0.05.

**Figure:**
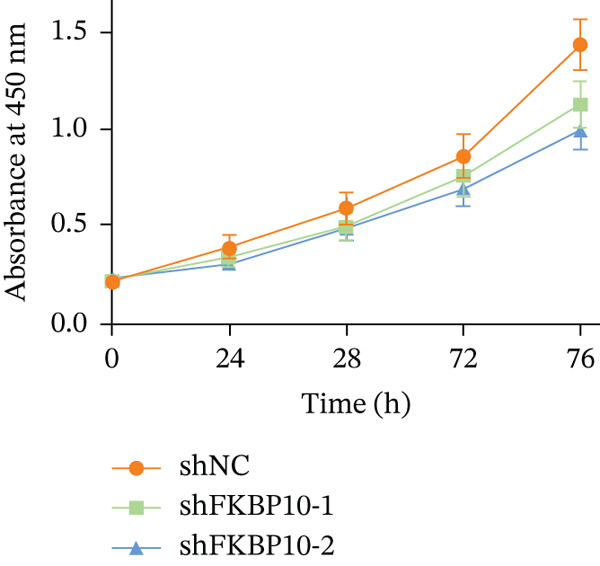
(a)

**Figure:**
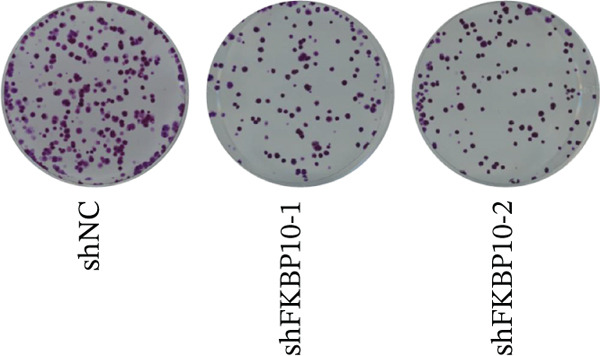
(b)

**Figure:**
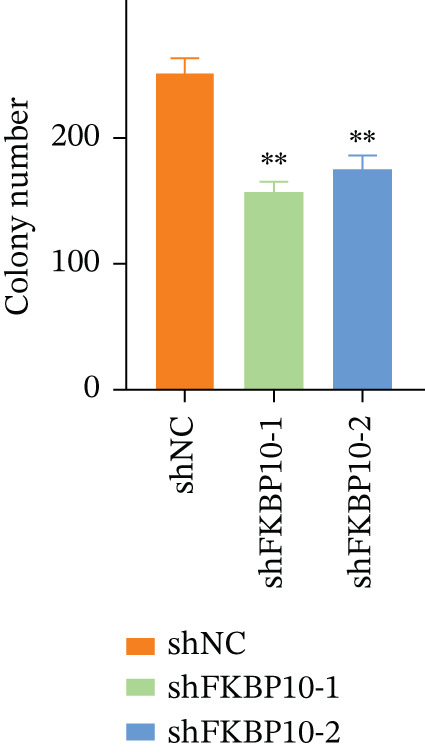
(c)

**Figure:**
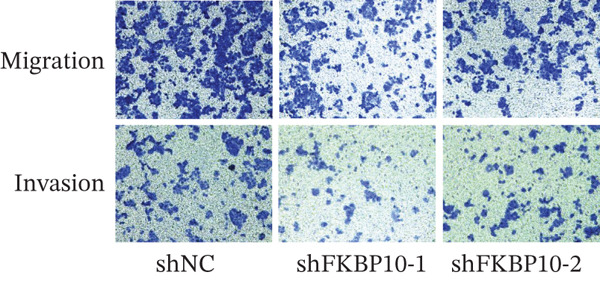
(d)

**Figure:**
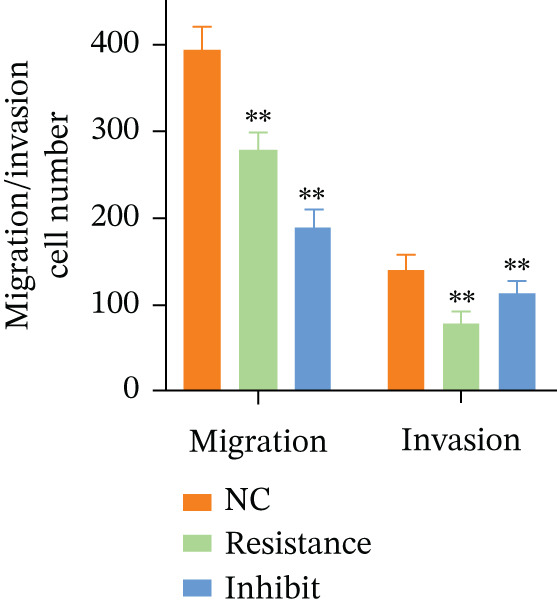
(e)

### 3.11. FKBP10 Knockdown Suppresses CRC Cell Migration and Invasion

Given the association of FKBP10 with ECM remodeling pathways, we investigated its role in cell migration and invasion using Transwell assays. FKBP10 knockdown significantly reduced cell migration, with shFKBP10‐1 and shFKBP10‐2 decreasing migrated cell numbers by approximately 30% compared to controls (Figure 8d,e, left panel, *p* < 0.01). Matrigel invasion assays revealed even more pronounced effects, with shFKBP10‐1 and shFKBP10‐2 reducing invasion by approximately 50% (Figure 8d,e, right panel, *p* < 0.01). These results demonstrate that FKBP10 promotes CRC cell migration and invasion, potentially through its role in collagen processing and ECM remodeling, which are critical for creating invasion‐permissive microenvironments.

### 3.12. FKBP10 Knockdown Markedly Enhances Radiosensitivity

To directly test whether FKBP10 contributes to radiotherapy resistance, we performed clonogenic survival assays following graded radiation doses (0, 4, 6, and 8 Gy). FKBP10 knockdown significantly enhanced radiosensitivity at all radiation doses tested (Figure 9a,b). At 4 Gy, shFKBP10‐2 cells showed 18% reduction in surviving fraction compared to control (*p* < 0.05). At 6 Gy, the reduction was 28% (*p* < 0.01), and at 8 Gy, the reduction reached 48% (*p* < 0.01). These dose‐dependent effects demonstrate that FKBP10 is a critical mediator of radiotherapy resistance and its inhibition substantially increases tumor cell killing by radiation.

Figure 9FKBP10 knockdown enhances radiosensitivity and increases radiation‐induced DNA damage. (a) Representative images of colony formation assays following radiation exposure (0, 4, 6, and 8 Gy). shFKBP10‐2 cells formed fewer colonies than shNC control cells at all radiation doses. (b) Clonogenic survival curves. Colony‐forming efficiency was normalized to untreated controls (0 Gy). FKBP10 knockdown (shFKBP10‐2, blue line) significantly enhanced radiosensitivity compared to control (shNC, orange line) at all doses tested (4 Gy:  ^∗^ *p* < 0.05; 6 Gy and 8 Gy:  ^∗∗^ *p* < 0.01). Data are mean ± SD from three independent experiments. (c) Representative immunofluorescence images of *γ*H2AX foci (green) in shNC and shFKBP10‐2 cells with or without 4 Gy irradiation. Cells were fixed 1 h postirradiation for *γ*H2AX immunofluorescence staining. Nuclei were counterstained with DAPI (blue). Merge panels show colocalization. shFKBP10‐2 cells exhibited more *γ*H2AX foci following irradiation, indicating increased DNA damage. (d) Representative comet assay images showing DNA damage in shNC and shFKBP10‐2 cells at 0 and 4 Gy. Cells were harvested immediately after irradiation for comet assay analysis. Increased tail DNA (red fluorescence) indicates greater DNA strand breaks. shFKBP10‐2 cells showed dramatically longer comet tails after 4 Gy irradiation. (e) Quantification of comet assay tail DNA percentage. Under basal conditions (0 Gy), shFKBP10‐2 cells showed slightly higher tail DNA than shNC (13% vs. 6%,  ^∗^ *p* < 0.05). Following 4 Gy irradiation, shFKBP10‐2 cells exhibited significantly higher tail DNA (43% vs. 22%,  ^∗∗^ *p* < 0.01), indicating impaired DNA damage repair or enhanced radiation‐induced damage. Data are mean ± SD from at least 50 cells per condition.

**Figure:**
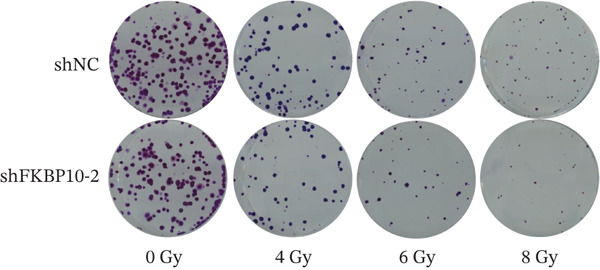
(a)

**Figure:**
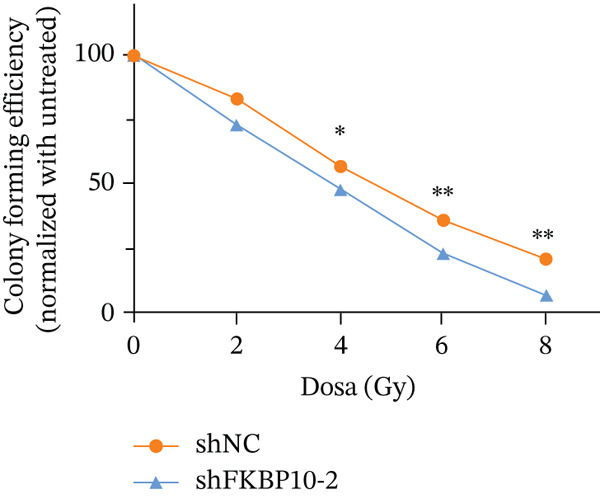
(b)

**Figure:**
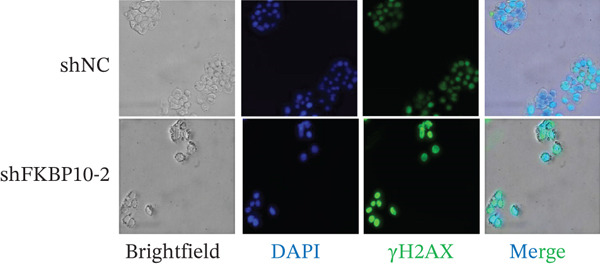
(c)

**Figure:**
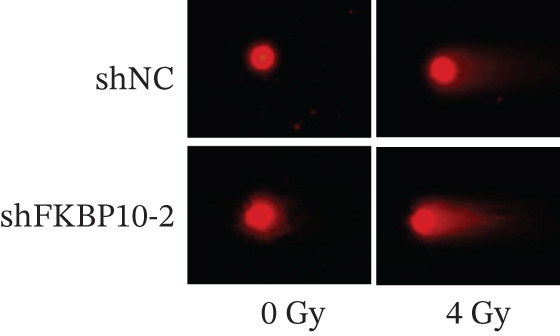
(d)

**Figure:**
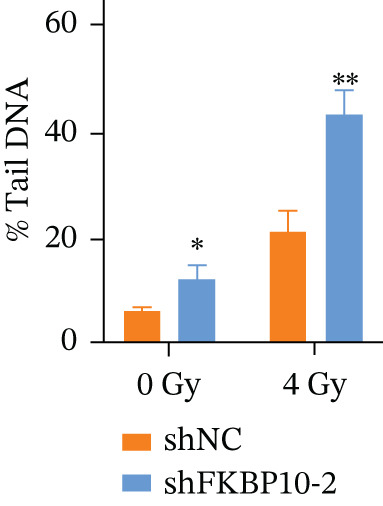
(e)

### 3.13. FKBP10 Knockdown Increases Radiation‐Induced DNA Damage

To elucidate the mechanism by which FKBP10 knockdown enhances radiosensitivity, we examined DNA damage responses. We first assessed *γ*H2AX foci formation, a sensitive marker of DNA double‐strand breaks, by immunofluorescence. Without irradiation, both control and FKBP10 knockdown cells showed minimal *γ*H2AX foci. Following 4 Gy irradiation, shFKBP10‐2 cells exhibited significantly more *γ*H2AX‐positive cells and higher numbers of foci per cell compared to control cells (Figure 9c). Comet assays, which detect DNA strand breaks, corroborated these findings. Under basal conditions (0 Gy), shNC and shFKBP10‐2 cells showed similarly low levels of DNA damage (tail DNA 6% vs. 13%, *p* < 0.05) (Figure 9d,e). Following 4 Gy irradiation, both groups showed increased DNA damage, but shFKBP10‐2 cells exhibited dramatically higher tail DNA percentage (43% vs. 22% in controls, *p* < 0.01) (Figure 9e). These results indicate that FKBP10 knockdown impairs DNA damage repair or enhances radiation‐induced DNA damage, leading to increased cell killing and radiosensitivity. The increased DNA damage in FKBP10 knockdown cells suggests that FKBP10 may contribute to radioresistance through mechanisms involving DNA repair capacity or protection from radiation‐induced genotoxic stress.

## 4. Discussion

In this comprehensive study, we integrated multiomics bioinformatics analyses with rigorous experimental validation to identify and characterize FKBP10 as a CAF‐derived biomarker of radiotherapy resistance in CRC. Our findings establish FKBP10 as a biomarker that not only predicts treatment response but also serves as a functional therapeutic target, as its inhibition significantly enhances radiosensitivity. This work exemplifies a translational pipeline from multiomics discovery to experimental validation, addressing a critical gap in biomarker research and providing actionable insights for precision oncology. The identification of FKBP10 as the sole gene achieving statistical significance across two independent patient cohorts, generated on different microarray platforms and involving different treatment regimens, is particularly compelling. While 48 genes were differentially expressed in the discovery cohort, only FKBP10 demonstrated robust and consistent upregulation in both discovery and validation cohorts. This cross‐cohort, cross‐platform validation substantially strengthens confidence in FKBP10 as a genuine biological signal rather than a technical artifact or cohort‐specific finding. A validation rate of 43.8% for directional consistency reflects inherent challenges in biomarker validation, including differences in patient populations, treatment protocols, and statistical power. Against this backdrop, FKBP10′s consistent performance underscores its biological and clinical relevance.

Several specific clinical and technical differences between the discovery and validation cohorts merit discussion to contextualize the 43.8% directional consistency rate. The discovery cohort (GSE87211, *n* = 200) comprised CRC patients treated with radiotherapy alone, while the validation cohort (GSE46862, *n* = 58) included locally advanced rectal cancer patients receiving neoadjuvant chemoradiotherapy with concurrent 5‐fluorouracil‐based chemotherapy. The addition of chemotherapy introduces additional pharmacological selection pressure that may alter gene expression profiles independently of radiation response, potentially explaining the discordance of many candidate genes. Furthermore, the two cohorts were profiled on different Affymetrix platforms (U133 Plus 2.0 vs. Gene 1.0 ST Array), which differ substantially in probe design, coverage, and sensitivity. The discovery cohort also had a larger sample size and a higher proportion of radioresistant patients (72.5% vs. approximately 65%), which may affect statistical power and effect size estimates. Despite these considerable sources of heterogeneity—different treatment regimens, platforms, patient populations, and sample sizes—FKBP10 remained the sole gene achieving statistical significance in both cohorts, further underscoring its robustness as a biologically meaningful biomarker.

Moreover, FKBP10 demonstrated functional coherence across multiple analytical layers. It was not merely a statistically significant gene but was also prominently featured in pathway enrichment analyses (ECM organization and collagen formation), exhibited cell‐type‐specific expression patterns aligned with its molecular function (CAF expression), and showed consistent upregulation in experimentally derived radioresistant cell lines. This convergent evidence from computational and experimental approaches strengthens the validity of FKBP10 as a radiotherapy resistance biomarker and exemplifies best practices in multiomics biomarker research. A critical limitation of many multiomics biomarker studies is the lack of experimental validation, which hinders clinical translation. Our study addresses this gap by systematically validating FKBP10 through multiple experimental approaches. We first confirmed that FKBP10 is upregulated in radiotherapy‐resistant CRC cell lines established through chronic fractionated irradiation, recapitulating the clinical observation. We then demonstrated through loss‐of‐function experiments that FKBP10 is not merely a biomarker but a functional mediator of radiotherapy resistance. FKBP10 knockdown reduced cell proliferation, colony formation, migration, and invasion—phenotypes associated with aggressive, therapy‐resistant cancers. Most critically, FKBP10 knockdown markedly enhanced radiosensitivity, as evidenced by reduced clonogenic survival and increased radiation‐induced DNA damage.

These experimental findings transform FKBP10 from a correlative biomarker to a causal contributor to radiotherapy resistance, thereby establishing it as a potential therapeutic target. This progression from computational discovery to functional validation represents a crucial step toward clinical translation, as functional validation provides mechanistic insights and therapeutic rationale that purely correlative biomarkers lack. Single‐cell RNA sequencing analysis revealed that FKBP10 is predominantly expressed in stromal cells, specifically CAFs, rather than tumor epithelial cells. This finding is significant for several reasons. First, it indicates that radiotherapy resistance is not solely a tumor cell–intrinsic property but involves active contributions from the stromal compartment. CAFs are known to secrete ECM proteins, remodel the stromal matrix, and produce growth factors that support tumor cell survival and proliferation [25]. FKBP10, as a collagen‐specific molecular chaperone, facilitates proper folding and assembly of fibrillar collagens (Types I, II, and III), enabling CAFs to construct a dense, protective stromal matrix [26, 27].

Second, the dense fibrotic stroma created by CAF‐derived FKBP10 and associated collagen deposition may contribute to radiotherapy resistance through multiple mechanisms: (1) physical barrier effects, limiting radiation‐induced reactive oxygen species (ROS) diffusion; (2) creation of hypoxic microenvironments through reduced oxygen diffusion, thereby decreasing ROS‐mediated DNA damage; (3) activation of mechanotransduction and integrin signaling pathways that promote survival and antiapoptotic signaling in tumor cells; and (4) sequestration of growth factors and cytokines in the ECM that continuously support tumor cell survival. Our pathway enrichment analyses consistently highlighted ECM organization, collagen formation, focal adhesion, and integrin signaling, supporting these mechanisms.

It is important to note that CAFs represent a highly heterogeneous population comprising distinct subtypes with different functional properties. In CRC, major CAF subtypes include myofibroblastic CAFs (myCAFs), characterized by high expression of *α*‐SMA (ACTA2), fibrillar collagens (COL1A1 and COL3A1), and ECM remodeling enzymes (lysyl oxidase [LOX] and LOXL2), and inflammatory CAFs (iCAFs), characterized by secretion of cytokines and chemokines (IL‐6, CXCL1, and CXCL12). Given FKBP10′s well‐characterized function as a collagen‐specific molecular chaperone that facilitates proper folding and cross‐linking of fibrillar collagens (Types I, III, and V), FKBP10‐expressing stromal cells most likely correspond to the myCAF subtype. This is consistent with our pathway enrichment results showing prominent enrichment of collagen fibril organization, ECM–receptor interaction, and focal adhesion pathways in radioresistant tumors. myCAFs are recognized as the primary producers of the dense desmoplastic stroma in CRC, and FKBP10‐positive myCAFs likely create a protective stromal niche that shields tumor cells from radiation‐induced damage through increased collagen density, reduced oxygen diffusion, and activation of integrin‐mediated survival signaling. Although the current publicly available scRNA‐seq dataset (GSE132465) does not provide sufficient resolution for robust CAF subclustering due to the limited number of stromal cells (*n* = 5933), future studies employing dedicated fibroblast‐enriched single‐cell profiling or spatial transcriptomics approaches could definitively assign FKBP10 expression to specific CAF subtypes and map their spatial relationships with tumor cells.

An important consideration is the dual expression of FKBP10 in both CAFs (51.5% of stromal cells) and tumor epithelial cells (23.2% of epithelial cells). Our functional experiments, performed in CRC epithelial cell lines (HT29 and SW480) in the absence of a CAF compartment, demonstrate that tumor cell–intrinsic FKBP10 contributes to radioresistance through cell‐autonomous mechanisms. This epithelial FKBP10 may promote resistance through autocrine ECM remodeling (pericellular collagen deposition and secretion that activates integrin signaling), intracellular collagen processing that affects ER homeostasis and stress responses, and potential cross‐talk between ER stress pathways and DNA damage response machinery. In the in vivo TME, FKBP10 likely mediates radioresistance through both paracrine mechanisms (CAF‐derived FKBP10 remodeling the stromal matrix) and autocrine mechanisms (tumor cell–intrinsic FKBP10 promoting cell survival). The combined effect of both sources is expected to be more pronounced than either alone, suggesting that our in vitro results may underestimate the total contribution of FKBP10 to radioresistance in clinical tumors. Future studies employing CAF–tumor coculture systems and conditioned media experiments from FKBP10‐overexpressing fibroblasts will be essential to dissect the relative contributions of paracrine versus autocrine FKBP10 signaling.

Third, stromal expression of FKBP10 suggests that targeting CAFs or ECM remodeling pathways could be a viable strategy to enhance radiosensitivity. Unlike targeting tumor cells directly, which often leads to resistance through clonal selection, disrupting tumor‐stromal interactions may alter the microenvironment in ways that make tumor cells more vulnerable to radiation. Our experimental results support this concept: Even in tumor cell lines cultured in vitro (where CAFs are absent), FKBP10 knockdown enhanced radiosensitivity, suggesting that tumor cells may also express FKBP10 (as shown by single‐cell data: 23.2% of epithelial cells) and that even tumor cell–intrinsic FKBP10 contributes to resistance, possibly through autocrine ECM remodeling or intracellular collagen processing. Our mechanistic experiments revealed that FKBP10 knockdown significantly increases radiation‐induced DNA damage, as demonstrated by elevated *γ*H2AX foci formation and increased DNA strand breaks in comet assays. These findings suggest that FKBP10 may contribute to radioresistance through mechanisms involving DNA damage repair capacity or protection from radiation‐induced genotoxic stress. While FKBP10 is classically known as an endoplasmic reticulum–resident collagen chaperone, emerging evidence suggests that FKBP proteins can have multifunctional roles, including regulation of DNA repair pathways and stress responses [28, 29].

Several mechanisms may explain how FKBP10 influences DNA damage responses: (1) ECM‐mediated signaling: FKBP10‐mediated collagen deposition activates integrin‐FAK‐PI3K/Akt signaling, which upregulates DNA repair proteins including DNA‐PKcs and RAD51, as well as antiapoptotic factors such as BCL‐2 and survivin [30]; (2) stress response pathways: FKBP10 may participate in unfolded protein response (UPR) or endoplasmic reticulum stress responses that cross‐talk with DNA damage response pathways through shared mediators such as ATF4 and NRF2; (3) TGF‐*β* pathway cross‐talk: FKBP10 has been reported to interact with and stabilize Type I TGF‐*β* receptor (TGFBR1), thereby promoting TGF‐*β* signaling which activates ATM/ATR‐mediated DNA repair through SMAD‐dependent transcription of key repair genes (BRCA1 and RAD51); (4) protein–protein interaction network analysis using the STRING database reveals that FKBP10 directly interacts with collagen processing enzymes (PLOD1, PLOD2, and SERPINH1) and is functionally connected to TGF‐*β* signaling (TGFBR1 and SMAD2/3) and integrin signaling networks (ITGB1 and FAK/PTK2), providing a molecular framework linking FKBP10′s chaperone function to downstream survival and DNA repair signaling. Despite these plausible mechanistic connections, the precise molecular pathway by which FKBP10 influences nuclear DNA repair remains to be fully elucidated [31]. Future studies employing DNA repair reporter assays, chromatin immunoprecipitation, and proteomic analyses of FKBP10‐interacting proteins will be needed to fully elucidate these mechanisms. Our findings have important implications for clinical translation. First, FKBP10 expression could be developed as a predictive biomarker to stratify patients for radiotherapy. Patients with high FKBP10 expression (particularly in stromal compartments, as assessed by immunohistochemistry [IHC] or spatial transcriptomics) may be less likely to benefit from standard‐dose radiotherapy and could be candidates for dose escalation, alternative treatment modalities, or enrollment in clinical trials of radiosensitizing agents. Conversely, patients with low FKBP10 expression may achieve favorable responses with standard radiotherapy, potentially allowing treatment de‐escalation and reduced toxicity.

Second, FKBP10 represents a potential therapeutic target for combination strategies to enhance radiosensitivity. Pharmacological inhibition of FKBP10 or downstream ECM remodeling pathways could be combined with radiotherapy to improve treatment efficacy. While specific FKBP10 inhibitors are not currently available, several strategies could be pursued: (1) repurposing existing FKBP inhibitors (e.g., FK506/tacrolimus, though these primarily target FKBP12) [32], (2) targeting downstream effectors of FKBP10, such as LOX or other collagen cross‐linking enzymes, for which inhibitors are in preclinical/clinical development [33], (3) targeting CAF activation pathways (e.g., TGF‐*β* inhibitors and FAP inhibitors) to reduce FKBP10 expression [30], and (4) developing novel small‐molecule or antibody‐based FKBP10 inhibitors through structure‐based drug design.

Third, our study demonstrates the value of multiomics approaches in identifying nonobvious therapeutic targets. Traditional radiotherapy biomarker research has focused on tumor cell–intrinsic factors (e.g., radiotherapy biomarker and DNA repair genes) [10]. Our unbiased transcriptomics approach revealed that stromal factors, particularly CAF‐derived FKBP10, play critical roles in radiotherapy resistance. This finding expands the therapeutic target space and highlights the importance of considering the TME in precision oncology strategies. Supporting the clinical relevance of our transcriptomics findings, publicly available IHC data from the Human Protein Atlas (HPA; http://www.proteinatlas.org) confirm that FKBP10 protein is expressed in CRC tissue, with notable staining detected in stromal and fibroblastic cells surrounding tumor glands. This protein‐level evidence corroborates our single‐cell RNA sequencing observation that FKBP10 is predominantly expressed in the stromal compartment and provides independent validation at the protein level. While formal quantitative IHC comparing FKBP10 expression in radioresistant versus radiosensitive patient tissues with validated antibodies and standardized scoring criteria remains an important future step for clinical translation, the consistency between our transcriptomics data and publicly available protein expression data strengthens the biological plausibility of FKBP10 as a stromal biomarker of radiotherapy resistance. This study directly addresses themes central to the development and implementation of multiomics biomarkers in clinical practice. We demonstrate a rigorous validation framework that progresses from large‐scale discovery, through independent cohort validation and single‐cell resolution analyses, to functional experimental validation. This multilayered validation approach is essential for establishing the clinical utility of biomarkers, which is a prerequisite for health technology assessment (HTA) and reimbursement decisions [34].

Furthermore, our work highlights the importance of functional validation in biomarker development. While many multiomics studies generate lists of candidate biomarkers, few demonstrate that these biomarkers are mechanistically linked to disease outcomes or represent actionable therapeutic targets. By showing that FKBP10 inhibition functionally enhances radiosensitivity, we provide a rationale for clinical development that goes beyond correlation, addressing a key criterion for HTA evaluation: evidence of clinical impact.

Our findings also underscore the need for appropriate bioinformatics infrastructure, workforce training, and interdisciplinary collaboration—themes emphasized in the special issue rationale. The integration of bulk transcriptomics, single‐cell sequencing, pathway analysis, and experimental validation required expertise spanning bioinformatics, molecular biology, and translational oncology. Such interdisciplinary approaches are essential for realizing the potential of multiomics biomarkers to improve patient outcomes. Several limitations should be acknowledged. First, this study is retrospective and hypothesis‐generating. Prospective validation in well‐annotated, multi‐institutional patient cohorts is required before clinical implementation. Ideally, FKBP10 expression should be assessed in pretreatment biopsies from patients enrolled in radiotherapy trials, with subsequent correlation to pathological response, survival outcomes, and quality of life metrics.

Second, while we demonstrated that FKBP10 knockdown enhances radiosensitivity in vitro, in vivo validation is needed to confirm these findings in the complex TME. Patient‐derived xenografts (PDXs) that preserve stromal components, or genetically engineered mouse models with intact CAF populations, would be ideal for testing FKBP10‐targeted therapies in combination with radiotherapy.

Third, our mechanistic understanding of how FKBP10 influences DNA damage responses remains incomplete. Detailed mechanistic studies employing DNA repair reporter assays, live cell imaging of DNA damage dynamics, proteomic identification of FKBP10‐interacting proteins, and investigation of specific DNA repair pathways (homologous recombination, nonhomologous end joining, and base excision repair) are warranted.

Fourth, our experimental validation was conducted in CRC cell lines cultured as monolayers, which do not fully recapitulate the 3D architecture and heterotypic interactions of tumors. In particular, while our knockdown experiments demonstrate the contribution of tumor cell–intrinsic FKBP10 to radioresistance, they do not directly test the paracrine mechanism of CAF‐derived FKBP10. Future studies should employ 3D organoid cultures, tumor‐stromal coculture systems, and, critically, conditioned media experiments using FKBP10‐overexpressing fibroblasts to determine whether extracellular FKBP10 can induce radioresistance in CRC cells through paracrine signaling. Such experiments would bridge the gap between our single‐cell findings identifying CAFs as the primary cellular source and the functional effects observed in epithelial cell lines.

Fifth, we did not incorporate genomic alterations, DNA methylation, or posttranscriptional regulatory mechanisms, which may also contribute to radiotherapy resistance and FKBP10 regulation. Integrative multiomics analyses incorporating these layers could provide more comprehensive insights.

Finally, clinical translation will require the development of companion diagnostics to assess FKBP10 expression. IHC assays with validated antibodies and standardized scoring criteria, or potentially spatial transcriptomics approaches, would be needed to quantify FKBP10 in tumor and stromal compartments in clinical samples.

## 5. Conclusion

This study demonstrates that FKBP10 is a robust, cross‐cohort validated biomarker of radiotherapy resistance in CRC. Through integrative multiomics analysis spanning bulk transcriptomics, single‐cell RNA sequencing, machine learning modeling, and comprehensive experimental validation, we established that FKBP10 is predominantly expressed in CAFs and functionally contributes to radioresistance. FKBP10 knockdown significantly inhibited CRC cell proliferation, colony formation, migration, and invasion, while markedly enhancing radiosensitivity through increased radiation‐induced DNA damage. These findings position FKBP10 as both a predictive biomarker for patient stratification and a promising therapeutic target for combination strategies aimed at overcoming radiotherapy resistance. Future prospective clinical validation and mechanistic studies will be essential to translate these findings into improved treatment paradigms for CRC patients.

## Author Contributions

Fu Xinmo conceived and designed the study, led the investigation and methodology, performed formal analyses and machine learning modeling, and drafted the initial manuscript; Bai Minghua carried out key data analyses and visualization, conducted pathway enrichment/survival and scRNA‐seq integration, and cowrote and critically revised the manuscript; Qiu Xu conducted experimental validation, assisted with methodology optimization, and contributed to data interpretation; Li Weiwei handled data curation and sample management and assisted with routine experiments; Wang Lin operated the irradiation system, supported cell culture and reagent preparation, and helped with experimental logistics; Dai Qinghui provided resources and technical support and assisted with quality control; Wang Rui and Zhu Ji served as co‐corresponding authors, supervising the project, providing resources and funding, overseeing methodology and quality control, interpreting results, and offering strategic direction and critical revisions. Fu Xinmo, Bai Minghua, and Qiu Xu contributed equally to this work and are considered co‐first authors.

## Funding

This work was supported by the National Natural Science Foundation of China (10.13039/501100001809, 82574019), the Jiaxing Key Research and Development Program of the Chinese Academy of Sciences (2024BZ20004), the National Health Commission Research Foundation (WKJ‐ZJ‐2305), and the Beijing Xisike Clinical Oncology Research Foundation (Y‐Gilead2024‐PT‐0092).

## Conflicts of Interest

The authors declare no conflicts of interest.

## Supporting information


**Supporting Information** Additional supporting information can be found online in the Supporting Information section. Table S1: Differentially expressed genes in radioresistant versus radiosensitive CRC.

## Data Availability

All gene expression datasets analyzed in this study are publicly available from the Gene Expression Omnibus (GEO) at http://www.ncbi.nlm.nih.gov/geo/ under Accession Numbers GSE87211, GSE46862, and GSE132465.
